# Recycling Potential of SLS-Degraded PA12 Powder for Melt-Based Manufacturing: Insights Resulting from the Advanced Crystallization Modeling

**DOI:** 10.1021/acsomega.6c03917

**Published:** 2026-07-15

**Authors:** Roman Svoboda, Jakub Vlachynský, David Jaška, Jana Machotová, Jana Navrátilová

**Affiliations:** † Department of Physical Chemistry, Faculty of Chemical Technology, University of Pardubice, Studentská 573, 532 10 Pardubice, Czech Republic; ‡ Department of Polymer Engineering, Faculty of Technology, 48362 Tomas Bata University in Zlín, Vavrečkova 5669, 760 01 Zlín, Czech Republic; § Institute of Chemistry and Technology of Macromolecular Materials, Faculty of Chemical Technology, University of Pardubice, Studentská 573, 532 10 Pardubice, Czech Republic

## Abstract

Selective laser sintering (SLS) of polyamide 12 (PA12) generates large quantities of thermally aged powder whose altered crystallization behavior limits its complete reuse in additive manufacturing. In the present study, the feasibility of repurposing SLS-degraded PA12 powder in conventional melt-processing techniques is investigated. Thermo-analytical, diffraction, spectroscopic, and microscopic techniques were employed to investigate the crystallization kinetics, crystal morphology, and mechanical properties of PA12 materials prepared from different SLS-processed powder grades, namely nondegraded (as purchased), degraded (recycled), and mixed (blended) PA12 powders. The mixed material (made from nondegraded and degraded powders in the weight ratio of 25/75) exhibited markedly slower crystallization, especially during isothermal and slow-cooling conditions, attributed to molecular incompatibility and impaired cocrystallization between long, irregular degraded chains and shorter nondegraded chains. Advanced kinetic modeling using the temperature-dependent M-catalytic Hoffman–Lauritzen framework confirmed increased nucleation barriers and distinct shifts in autocatalytic and growth-related kinetic parameters. Hot-stage microscopy further revealed distinct differences in the crystal growth kinetics, explaining the variable lamellae packing within forming spherulites. Mechanical properties of the melt-processed PA12 materials significantly differed below the glass transition temperature (*T*_g_) – stiffness of the degraded material increased, while it decreased for the mixture. On the contrary, above *T*_g_, the mechanical behavior was practically identical for the nondegraded material and for the blend with 75% of degraded material, which introduces an interesting alternative utilizing the SLS-processed PA12 powders in melting-based technologies, and provides guidance for optimizing compositions to balance processability, cost-effectiveness, and performance of the corresponding recycling.

## Graphical abstract

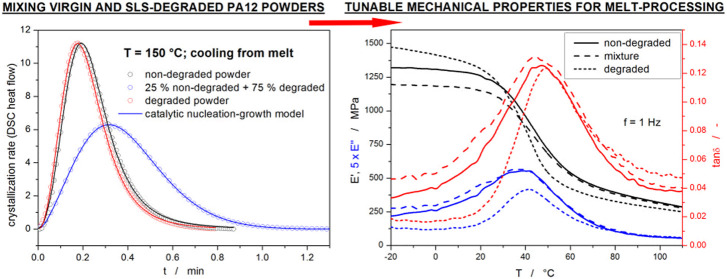

## 1 Introduction

Polyamide 12 (PA12), commonly known as nylon-12, is a thermoplastic polymer highly valued in various industries due to its exceptional physical properties. The toughness, durability, and abrasion resistance of PA12 (attributed to the presence of amide groups in its molecular structure and the hydrogen bonds between adjacent molecular chains) are crucial, e.g., for automotive and aerospace industries.^1,2^ PA12 also exhibits excellent barrier properties against water vapor, oxygen, and oils, comparable to traditional polymers used in packaging and technical applications.^2,3^ The favorable physicochemical and mechanical properties identify PA12 as a biocompatible material suitable for use in bone tissue engineering and other medical applications.^4,5^ High thermal stability and processability of PA12 allow its utilization for the preparation of a large variety of composites, further massively expanding the applicability of this polymer; some of the recently featured additives include carbon fibers, nanoclays, silicon carbide (SiC), titanium dioxide (TiO_2_), aluminum oxide (Al_2_O_3_), boron nitride (BN), or hydroxyapatite (HA).^6–9^

One of the key technologies for the above-listed PA12-based applications is the selective laser sintering (SLS) 3D printing. This additive manufacturing technique involves using a laser beam, typically a CO_2_ laser, to selectively sinter powdered materials based on a three-dimensional computer-aided design model. The laser fuses the powdered material layer by layer, enabling the creation of three-dimensional objects with high precision and dimensional accuracy.^10^ In contrast to e.g., fused-deposition modeling (FDM), the SLS procedure utilizes a rather excessive amount of the raw powdered material that remains dormant during the printing process, serving as a support, externally reinforcing the 3D-printed object (typically, this fraction represents ∼ 90% of the powder involved in the SLS process).^11^ It is a customary practice to reuse/recycle this unused powder. However, as the typical printing times are in the order of hours, the unused powder is exposed to the prolonged periods of thermal stress (both from the heated bed of the printer and from the vicinity of the laser beam sintering the adjacent layers), which can lead to the alteration of its properties.^12,13^

This is indeed the case for the recycled PA12, as was demonstrated in several studies dealing with the partial degradation of the SLS-produced waste powder. As a consequence of the SLS processing, significant changes in the powder size, molecular weight, melt viscosity, crystallinity, and even the dominant polymorphic crystalline phase were reported in literature for the recycled PA12 powder,^14,15^ ultimately leading to the major variation of the mechanical properties of the printed objects.^16^ The recent review on this topic^17^ has drawn attention to a particularly important topic of the changes in the crystallization behavior of the SLS-degraded PA12 powder with regard to its reusability in the SLS process. Since PA12 is a semicrystalline polymer that always crystallizes to the maximum extent (crystallinity of 50–55%) during the cooling of the melt, even relatively small changes in the apparent crystallization kinetics (such as a shift of the crystallization temperature *T*_c_ by ∼ 2 °C, as reported in^17^) can translate into a significantly different polymorphic or morphological state of the forming crystalline phase. It has been further shown that working with mixtures of the virgin/nondegraded and SLS-degraded PA12 powders can often be beneficial, significantly improving the properties of the 3D-printed products in comparison with the utilization of only the SLS-degraded powder and economizing the virgin material.

In the present study (sequel to ref^17^), we explore the possibility of reusing the SLS-degraded powder outside of the SLS 3D printing process, during standard melting-based processes, e.g., injection/blow/rotational/compression molding or extrusion. Akin strategy, i.e., exploring the advantages/disadvantages of utilizing the mixture of the nondegraded and SLS-degraded PA12 powder, will be adopted. In particular, a novel advanced mathematical framework derived for the description of the crystallization kinetics (recently introduced in ref^18^) will be used to identify the origin of the differences in the crystallization behavior of the mixtures combining the nondegraded and SLS-degraded PA12 powders; the experimental exploration of these differences will be performed by means of differential scanning calorimetry, X-ray diffraction analysis, dynamic mechanical analysis, Raman spectroscopy, and microscopic techniques. The study aims to offer a perspective, including limitations, in the potential utilization of the SLS-degraded PA12 powder in the melting-based technological processes.

## 2 Experimental Section

The commercial-grade PA12 powder (PA 2200) was purchased from EOS GmbH (Germany). This powder was exposed to a series of industrial SLS procedures performed on an EOS P 396 PBF printer (EOS GmbH, Germany); on average, the powder was exposed to 5–10 cycles (duration of the cycle varied within the 3–10 h range), with the powder bed heated to 170 °C and the baseplate heated to 130 °C (more details are given in^17^). In addition to the nondegraded (as purchased) and SLS-degraded (recycled) powders, a mixture prepared from these powders by thorough manual mixing in the 1:3 ratio (25 w.% of the nondegraded powder and 75 w.% of the degraded powder) was investigated.

The bulk of the experimental measurements was performed by means of differential scanning calorimetry (DSC), using the Q2000 calorimeter (TA Instruments) equipped with an autosampler and RCS90 cooling accessory. The measurements were performed in the T-zero heat flow mode; the DSC was calibrated based on the melting temperatures and enthalpies of the In, Zn, and H_2_O standards. The PA12 powders were hermetically sealed in low-mass Al pans, i.e., the measurements were performed in the atmosphere of static air. The sample masses varied in the 3.1–3.3 mg range (determined with an accuracy of ± 0.05 mg). All measurements for the given type of PA12 powder were performed for a single sample, which ensured excellent accuracy and reproducibility of the measurements. All measurement series were once reproduced for all three samples (nondegraded powder, powder mixture, and degraded powder) to confirm the reproducibility and to verify that no thermal degradation occurred during the repeated melting/solidification cycling during the DSC experiments. It has to be noted that the initial melting of the powder during the first DSC heating scan led to the formation of a thin compact PA12 disc on the bottom of the DSC pan; thus, the properties of the PA12 material (as opposed to the “powder”) were mostly studied by this technique in the present paper. Nonetheless, these samples (probably due to their compact melted and solidified state) had no further tendency to degrade or to change their properties even during prolonged cycling in the 0–200 °C temperature range.

The following two types of temperature programs were applied during the DSC experiments:

(1) Cyclic nonisothermal experiments, where the sample was first annealed for 1 min at 200 °C, then it was cooled at a selected cooling rate q^–^ to 20 °C, and then, it was heated at 20 °C·min^–1^ back to 200 °C. These cycles were repeated (for the identical sample) with cooling rates q^–^ = 0.2, 0.5, 1, 2, 3, 5, 7, 10, 15, 30, and 50 °C·min^–1^, and heating rate q^+^ = 20 °C·min^–1^. Visual demonstration of this temperature program (for q^–^ = 5 °C·min^–1^) is shown in Figure 1A.
(2) Cyclic isothermal experiments, where the sample was first annealed for 1 min at 200 °C, then it was cooled ballistically (programmed rate of 100 °C·min^–1^; true rate ∼75 °C·min^–1^) to the selected annealing temperature *T*_a_, then a sufficiently long annealing followed to fully crystallize the sample, after that the sample was cooled at q^–^ = 10 °C·min^–1^ to 140 °C, and last, the sample was heated at 5 °C·min^–1^ to 200 °C. The annealing temperatures applied within the isothermal cycles were *T*_a_ = 150, 152.5, 155, 157.5, 160, 162.5, 165, 167.5, and 170 °C. A visual demonstration of one isothermal (*T*_a_ = 150 °C) cycle is shown in Figure 1B.

**Figure 1:**
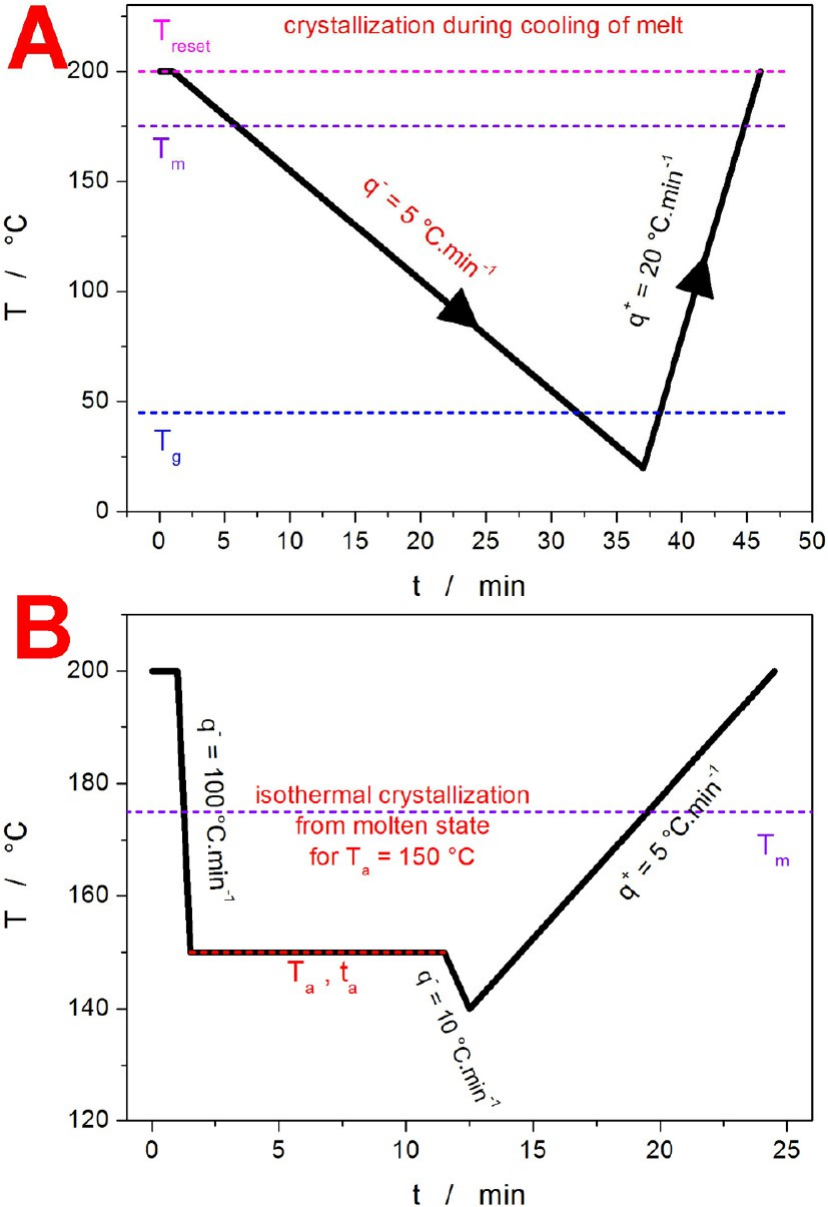
Temperature programs for the nonisothermal crystallization occurring during cooling (graph A) and isothermal crystallization followed by a polymorph-monitoring heating scan (graph B).

Apart from the DSC technique, additional measurements were performed for the melted and solidified materials made from three PA12 powder types (nondegraded, SLS-degraded, mixed). To assess the resistivity of PA12 materials against a high-temperature thermal degradation, thermogravimetry (TGA) paired with differential thermal analysis (DTA) was employed, using the STA (TGA) 449 F5 Jupiter instrument (Netzsch) equipped with a DSC/TG holder. The samples for these measurements were prepared in DSC by melting the material at 200 °C (above its melting point *T*_m_) for 1 min and then cooling it at 50 °C·min^–1^ to laboratory temperature. Two series of the TGA/DTA measurements were performed for each powder type, utilizing either an N_2_ or a synthetic air atmosphere purging the measurement chamber at 50 mL·min^–1^. The sample masses were approximately 5 mg (weighted accurately to 0.01 mg). The measurements were performed in the 30–600 °C temperature range at a heating rate q^+^ = 10 °C·min^–1^. Reproducibility of the TGA measurements was verified for the nondegraded sample under both atmospheres.

Structural information was obtained for the original powders as well as for the melted and solidified PA12 materials by means of Raman spectroscopy, using the DXR2 Raman microscope (Thermo Fisher Scientific) equipped with a 785 nm excitation diode laser (laser spot size 1.6 μm) and a CCD detector. The measurements were performed with 25 mW laser power focused on the sample, 5 s duration of a single scan, and 60 scans accumulated in one spectrum, in a spectral range of 50–3375 cm^–1^. Similar practice was also used for the X-ray diffraction (XRD) measurements, utilizing the wide-angle X-ray scattering (WAXS) on an XRDynamic 500 instrument (Anton Paar). The measurements were performed in the Bragg–Brentano geometry with a Primux 3000 sealed-tube X-ray source with a Cu anode and a Ni/C divergent beam multiplayer monochromator with a wavelength of λ = 0.154 nm in reflection mode. A range of diffraction angles was set to 2θ = 5–35° with a step size of 0.05°. The measurements were carried out using a low-temperature chamber TTK600 (Anton Paar), where the sample was heated from 50 to 230 °C at 50 °C·min^–1^, annealed at 230 °C for 3 min, and then cooled to 50 °C at either 0.1 or 50 °C·min^–1^. Diffraction patterns were taken at 50 °C on the original powder samples, then in the melt at 230 °C, and subsequently after cooling at a controlled rate to 50 °C. The total crystallinity (χ_c_) of the samples was calculated using the XRD analysis software (Anton Paar) using the fitted areas of the crystalline (*A*_c_) and amorphous (*A*_a_) parts as χ_c_ *=* (*A*_c_/*(A*_c_ *+ A*_a_)) × 100 (%).

Temperature-dependent morphology of the crystalline phase forming in the melted PA12 samples was investigated by means of optical microscopy. The initial screening was performed using the iScope PLMi (Euromex) optical microscope in the reflection mode, equipped with a series of ×4–×80 objectives and Moticam digital camera (with ×1 magnification adapter). The investigation of the crystal growth rate was then performed by using the Olympus BX41 polarizing optical microscope in transmission mode equipped with the Infinity2 digital camera. The samples were produced by pressing a small amount of powder between two cover glasses in a manual press at a temperature of 210 °C for 2 min. This produced thin films approximately 20 μm thick. The films, together with the cover glasses, were placed in a Linkam TP 94 heated stage, melted at a temperature of 230 °C for 3 min, and then cooled in a controlled manner at rates of 0.1 and 50 °C/min to a temperature of 50 °C. Micrographs of the original PA12 powders (before their melting) were taken using the VHX-7000 Digital 3D Microscope (Keyence International, Belgium).

The mechanical properties of the PA12 materials were investigated by means of dynamic mechanical analysis (DMA), using the DMA303 Eplexor device (Netzsch) in a tensile configuration with the span between the clamps of 10 mm. The samples for the DMA were prepared by compression molding in a manual press at 200 °C for 5 min, producing thin strips with 0.9 × 2.2 × 100 mm^3^ dimensions. The DMA measurements were then performed during a multifrequency iso-step temperature program: the sample was heated via a step-by-step sequence of isothermal annealings from −20 to 110 °C, with the step being 2 °C; the moduli were determined during each isothermal hold for the series of frequencies: 1, 2, 3, 5, and 10 Hz. The dynamic load was set to produce a constant deformation of 30 μm with the maximum force limit of 20 N. The static load was then calculated proportionally to the applied dynamic force with a factor of 1.3 (with the static strain limit being set to 10%). The reproducibility of the DMA data was confirmed for three different strips at 25 °C.

Regarding the experimental flowchart, it can be best explained on the basis of sample preparation. The experimental route started with the raw powders, for which the series of nonisothermal and isothermal DSC experiments (starting from the melt), TGA, and base optical microscopy (particle size) were performed. Then, the powders were melted and cooled either at 0.1 or at 50 °C·min^–1^ to prepare compact samples for the XRD, Raman spectroscopy, and optical microscopy (morphology, density, and size of the crystallites). Thin layers of molten compact PA12 material were used in hot-stage microscopy to visually monitor the crystal growth rates. Finally, the compression molding was used to prepare the DMA samples from different powder grades. Regarding the advanced processing, the kinetics of macroscopic crystallization and microscopic crystal growth were determined from the DSC and hot-stage microscopy data, respectively.

Note that images of selected experimental setups are included in the Supporting Information – Section S1.

## 3 Results and Discussion

This section will be split into three parts. In the first subsection, a fundamental characterization of the melted PA12 materials made from three PA12 powder types (nondegraded, degraded, mixed) will be presented, introducing the differences in their thermal, structural, morphological, and mechanical properties. In the second subsection, the novel advanced model for the polymers’ crystallization kinetics will be utilized to investigate the observed anomalous crystallization behavior of the PA12 material made from the powder mixture from the macroscopic perspective (based on the calorimetric measurements). The third subsection will then explore the crystallization behavior of PA12 materials from the microscopic point of view, utilizing the crystal growth and nucleation rate measurements performed using hot-stage optical microscopy.

### 3.1 Characterization of PA12 Materials Regarding the Powder Type

The bulk of the experimental data was obtained by means of nonisothermal and isothermal DSC measurements. Several typical examples of the raw DSC curves obtained for the nondegraded PA12 powder-based material are shown in Figure 2. It is immediately clear that with increasing cooling rate q^–^, the crystallization peak shifts to lower temperature (as a consequence of the nucleation and crystal growth processes being given less time to manifest), but also the polymorphic character of the formed crystalline phase changes. At very low q^–^, the α-phase polymorph (thermodynamically stable triclinic lattice; *T*_m_ ≈ 178–182 °C) is formed practically exclusively at temperatures just below the melting point *T*_m_. As q^–^ increases, a proportionally increasing amount of the metastable γ-phase (monoclinic lattice; pseudohexagonal chain packing; *T*_m_ ≈ 165–175 °C) forms, as evidenced by the manifesting melting prepeak during the consequent heating step.^19^ Although the exact amounts of the formed polymorphic phases cannot be determined due to the possible α→γ recrystallization induced by the melting of the metastable phase, the typically regular shape of both melting peaks occurring after the cooling at 10 °C·min^–1^ suggests that the possibly overlapping exothermic recrystallization signal is only a minor contribution to the overall heat flow.

**Figure 2:**
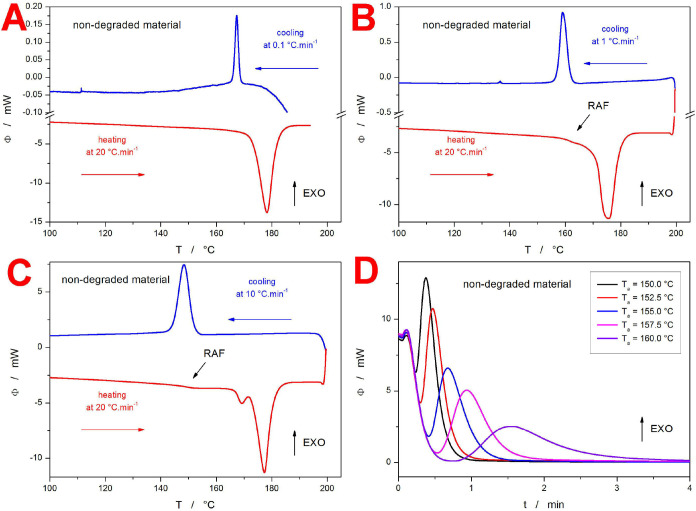
(A–C) Nonisothermal DSC cycles performed for the PA12 material originated from the nondegraded powder at different q^–^. (D) Selected isothermal DSC measurements (the annealing steps) performed for the PA12 material originated from the nondegraded powder. Exothermic effects evolve in the upward direction.

Worth noting is also the small endothermic signal on the heating curve occurring at the temperatures corresponding to the onset of the crystallization signal from the cooling step (160–165 °C in Figure 2B, and 145–154 °C in Figure 2C). The endothermic signal is associated with the release of the structural relaxation stress stored within the low-temperature phase of the rigid amorphous fraction (RAF)^20,21^ formed at the interface of the crystalline and fully amorphous phases. Note that the endothermic signal originates from a physicochemical background similar to that of the glass transition, manifesting for the amorphous polymeric phase.^21^ In addition to the nonisothermal data, several examples of the isothermal DSC signals obtained for the material made from the nondegraded PA12 powder are shown in Figure 2D—the lower the temperature, the higher the undercooling (defined as Δ*T* = *T*_m_ – T_a_) acting as a driving force for the nucleation and crystal growth processes. With increasing Δ*T*, the onset of the crystallization process shifts to earlier times, increasingly more overlapping with the instrumental artifact response associated with the heat flow change during the rapid cooling→isothermal annealing transition.

For the crystallization signals to be processable with regard to the description of their kinetics, their thermo-kinetic background (baseline signal) needs to be subtracted first. In the case of nonisothermal data, the default option is the physically meaningful tangential area-proportional baseline:^22^

1 $$B\left(T\right)=\left(1-\alpha \left(T\right)\right)\cdot \left(z_{0,r}+z_{1,r}T\right)+\alpha \left(T\right)\cdot \left(z_{0,p}+z_{1,p}\cdot \left(T_{f}-T\right)\right)$$

where B­(*T*) is the temperature dependence of the baseline curve, α is the degree of conversion (from the reactants/amorphous phase to the products/crystalline phase), *z*_0,r_ and *z*_1,r_ are the coefficients characterizing the tangent going through the starting point (in the reactants area), *z*_0,p_ and *z*_1,p_ are the coefficients characterizing the tangent going through the end point (in the products area), and *T*_f_ is the end point temperature. The full set of the nonisothermal DSC crystallization signals obtained for the PA12 material originated from the nondegraded powder (with the baselines already subtracted) is shown in Figure 3A,B. At low q^–^, the asymmetry of the crystallization peaks is quite similar, skewing to lower *T* (higher α), which is a characteristic^23^ typical for the Avrami nucleation–growth kinetics.^24–26^ As the q^–^ increases, the peak asymmetry changes from the negative asymmetry toward the symmetric or even positively asymmetric peaks (skewed to higher *T* and lower α). However, as shown in^27^ this does not necessarily indicate a deviation from the nucleation–growth kinetic mechanism but can only be a consequence of the temperature-dependent activation energy of the crystallization process. This possibility will be explored in detail in Section 3.2, which focuses on the macroscopic (DSC-monitored) crystallization kinetics.

**Figure 3:**
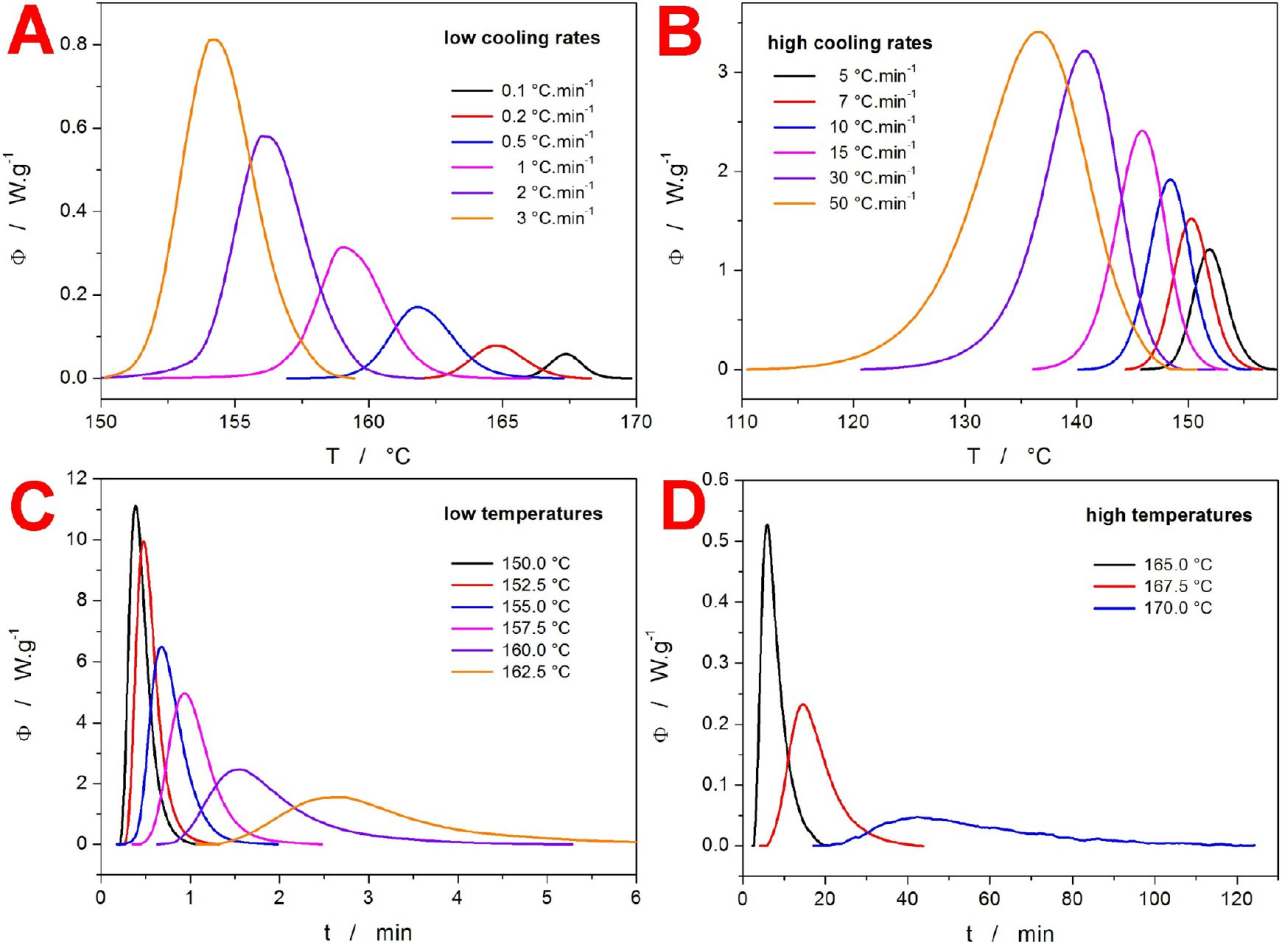
Full sets of the baseline-subtracted DSC data obtained for the PA12 material made of the nondegraded powder. Data were measured nonisothermally (graphs A and B; legends show cooling rates q^–^) and isothermally (graphs C and D; legends show annealing temperatures *T*_a_). Exothermic effects evolve in the upward direction.

The separation of the pure crystallization signal from the isothermal DSC data can efficiently utilize the almost perfect reproducibility and overlaps of the instrumental artifact signals obtained during isothermal experiments at different annealing temperatures (see Figure 2D). In the case of high isothermal temperatures, the instrumental artifact signal and the signal corresponding to the PA12 crystallization are fully separated (see, e.g., the curve for *T*_a_ = 160 °C in Figure 2D), and the instrumental artifact signal can thus be used as a baseline for the data curves, where the two signals are not fully separated. The full set of the isothermal crystallization signals obtained for PA12 material from the nondegraded powder is shown in Figure 3C,D.

As the main goal of the present research was to investigate the differences in the crystallization behavior of the melted PA12 powders with varying contents of the SLS-recycled admixture, identical experimental and baseline-processing procedures were also performed for the DSC data sets obtained for the material prepared from the degraded powder and for the materials made from the mixture of the nondegraded and degraded powders. Several example comparisons of the raw nonisothermal and isothermal DSC curves obtained for the three types of PA12 materials are shown in Figure 4. Concerning the nonisothermal data (Figure 4A–C), at moderate-to-high cooling rates, the crystallization tendency decreases with the content of the SLS-degraded powder. This is manifested as a decrease in both the crystallization temperature and the crystallization enthalpy. However, at very slow cooling (Figure 4A), the PA12 material originated from the mixture of the two powders (nondegraded + degraded) crystallizes slower than the materials made solely from the nondegraded or degraded powders, with its polymorphic profile being also slightly shifted in favor of the metastable γ-phase. In the case of the isothermal crystallization (Figure 4D–F), the crystallization of the mixture is at all temperatures much slower compared to the crystallization rate of the materials from sole powders. The base quantification of all DSC crystallization data, i.e., onset and peak crystallization temperatures (*T*_ons_, *T*_c_^p^), crystallization enthalpy Δ*H*_c_, extrapolated onset and peak temperatures of the melting process (*T*_m_^ons^, *T*_m_^p^), melting enthalpy ΔH_m_, induction time t_ind_, and time attributed to the maximum of the isothermal crystallization peak, are listed in Section S2.

**Figure 4:**
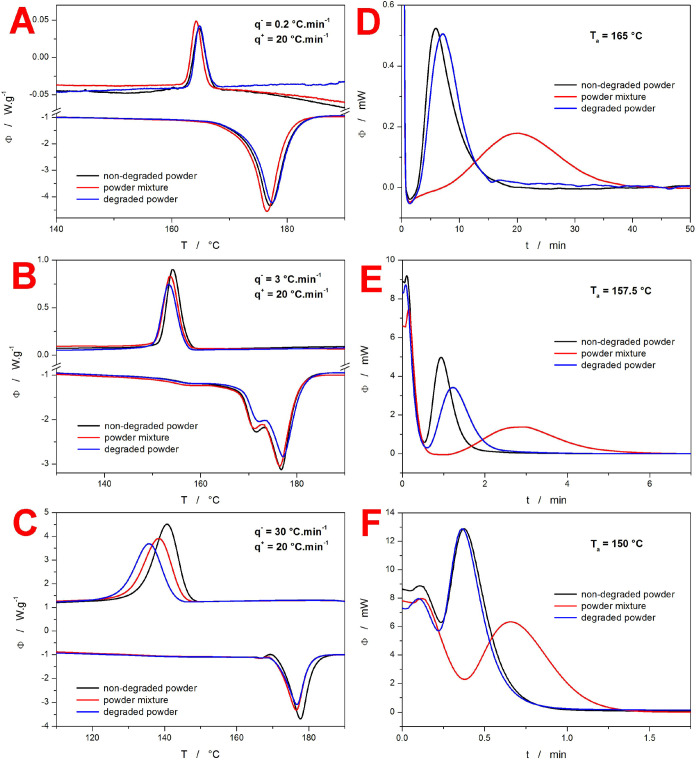
Example comparisons of the nonisothermal (graphs A–C) and isothermal (graphs D–F) DSC data obtained for the three types of PA12 materials (differing in the PA12 powder type). Exothermic effects evolve in the upward direction.

The noteworthy information provided by the DSC technique is the phenomenon of largely decreased crystallization rate in the case of the material made from the mix of nondegraded and SLS-degraded PA12 powders. By considering the conditions under which this effect occurs, the following explanation can be derived. The dominant rate-determining factor is certainly the nucleation process, which is crucial, especially during isothermal annealing (following the rapid quench) or very slow cooling. In both cases, the crystal growth process begins with a small number of nuclei and is therefore highly sensitive to the nucleation density and any factors that can alter it. Note that at higher cooling rates, the nucleation is more extensive due to the increased driving force (undercooling) and a larger number of accessible active growth sites due to the effective nucleation barrier and the critical diameter of the nuclei being largely lowered. During the SLS 3D printing, degradation of PA12 polymer occurs mainly due to the postcondensation reaction,^28^ increasing molar mass, polydispersity, and melt viscosity.^17^ Of course, other common degradation reactions, such as main-chain scission and thermo-oxidation, leading to changes in the molecular architecture of PA12 polymer chains, may also occur. However, at the temperatures of the SLS process, these reactions are considered to be less prevalent. Due to the changes in polymer structure (shorter linear polymer chains in the nondegraded powder vs longer chains of irregular structural arrangement in the degraded powder), the degraded powder exhibits decreased nucleation/crystallization rate and different crystal growth preferences (lamellar thickness) compared to the nondegraded powder. The reason for the powder mixture crystallizing significantly slower than the two basic powders is probably the molecular incompatibility of the two materials, which are, after melting, intimately intermixed. The chemical and structural irregularities in the degraded powder may result in mutually poor cocrystallization, disrupting the organization of the shorter, regular chains of the nondegraded material. This interference can lead to kinetic frustration, reducing the nucleation efficiency and early lamellar perfection.^29,30^

To further investigate the aspects/manifestations of the DSC-identified anomalous behavior of the mixture of the nondegraded and SLS-degraded PA12 powders, additional experimental techniques were used. For each powder, the samples were prepared by melting the given powder and cooling it at either 0.1 or 50 °C·min^–1^. The polymorphic characterization of the melted powders was done using the temperature-resolved XRD (see Figure 5). For each sample, three XRD diffraction patterns were taken: before melting (in the original powdered form), in the molten form at 230 °C (to verify the complete melting of the material), and after the cooling (solidified crystallized material at 50 °C). An example of such patterns is shown for the nondegraded sample cooled at 0.1 °C·min^–1^ in Figure 5A.

**Figure 5:**
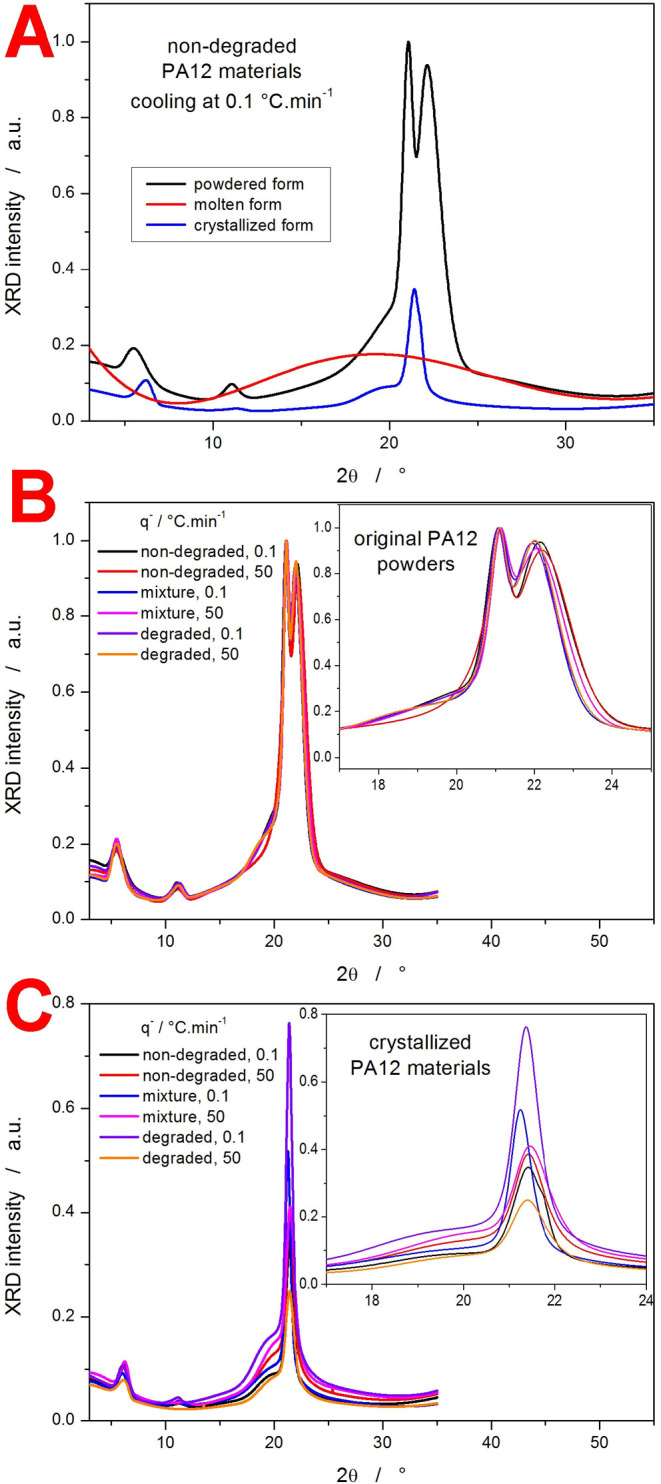
(A) XRD patterns obtained during the heating→cooling measurements of the nondegraded PA12 powder (the patterns correspond to the original powder, its melt, and the solidified material crystallized during the cooling of the melt at 0.1 °C·min^–1^). (B) XRD patterns obtained for the different types of original PA12 powders before the preparation of the melted materials presented in Figure 5C. (C) XRD patterns obtained for the set of the crystallized (solidified) materials made from different powder types at different cooling rates.

Since the XRD patterns obtained for the molten powders were collected only as a form of verification and contain no informational value, only the crystalline patterns will be discussed in the following text. As is shown in Figure 5B, all original powders were in the complex polymorphic composition: α-phase at 2θ approximately 6°(002), 20°(200), and 23°(010); γ-phase at 2θ approximately 6°(020), 12° (040), and 22°(001); and γ′-phase at 2θ approximately 6°(020) and 22°(001), with minimum XRD pattern differences between the individual powders (nondegraded vs degraded). After remelting and cooling, a γ-phase polymorph diffraction line is dominantly occurring at 21.4° 2θ with an α-phase shoulder in the 18–20.5° 2θ region.^31^ Worth noting is a conceptual agreement with the results reported in^14^, where a mixture of the α and γ phases is reported to form at temperatures considered in the present study, with the γ phase having the growth maximum shifted to slightly lower *T*. As can be seen in Figure 5C, the proportion between the α and γ signals remains roughly similar, confirming that in the present range of crystallization temperatures explored during cooling (∼130–170 °C), the proportion between the nucleation and growth rates of the two polymorphs remains similar. An apparent contrast arises from the isothermal data reported in^14^, where the significant formation of the γ phase is attributed only to the 80–140 °C range. But since these experiments were performed by means of (and modeled for) the rapid cooling at 1000 °C·s^–1^ in a flash DSC, the nucleation was largely suppressed during the cooling step, to which the polymorphic distribution is evidently very sensitive.

Generally, stronger XRD signals were obtained with decreasing cooling rate q^–^ (the crystalline phase formed at high *T*, with denser and more regular lamellae packing) and with increasing content of the degraded material in the original powder. However, it is necessary to take into account that the signal intensity depends on the quality of the sample surface, which was created in a temperature cell without the possibility of control. The calculated degrees of crystallinity χ_c_ are listed in Table 1. Interestingly, the XRD results show higher crystallinity for the degraded powder, while the results provided by DSC (see Tables S1–S3) show an opposite trend. This further testifies about the structural morphology of the crystalline phase. The melting enthalpy recorded by DSC identifies total crystalline mass fraction, which is sensitive to the crystal perfection and lamellar thickness, i.e., the total mass of the ideal crystalline phase. On the other hand, XRD monitors coherent diffraction intensity, identifying the fraction of the material giving a sufficient long-range order (typically, domain sizes ≥ ∼ 10 nm); the technique is thus less sensitive to small disordered and defective crystals. Since the SLS-degradation leads to molar mass and melt viscosity increase coupled with a higher number of structural irregularities in polymer chains, the chain folding is disrupted, and the lamellar thickness is reduced, resulting in lower specific enthalpy determined from the DSC data (= lower apparent crystallinity). Based on the XRD data, well-developed crystallites with better-ordered lamellae evidently grow at high temperatures during slow cooling. While intuitively correct, no evidence for this feature can be derived from the so far presented results, where only the changes in the nucleation processes were inferred and discussed (as opposed to the characteristics of the consequent crystal growth). For this main XRD trend, the confirmation of the physicochemical origin will be presented in Section 3.3, dealing with the microscopically monitored growth rate. Note that apart from the fundamental changes in the growth rate (resulting in better lamellae packing), further aspects can contribute to the higher apparent XRD crystallinity of the SLS-degraded PA12, e.g., reduced amorphous background scattering, preferred orientation, or polymorphic purity; none of these can, however, be unambiguously proven for the present data.

**Table 1:** Crystallinity χ_c_ (in %) Determined by XRD for the Original PA12 Powders and for PA12 Materials Crystallized at Different q^–^ from Melt

		crystallized from melt at	
sample	powder	50 °C·min^–1^	0.1 °C·min^–1^
PA12 nondegraded	56	35.4	49.5
PA12 mixture	61	46.8	64.5
PA12 degraded	64	50.6	67.1

Additional investigation of the differences between the PA12 materials, regarding the powder type, was done by means of Raman microscopy (see Figure 6A), where the Raman spectra corresponding to the above-described crystallized (solidified) materials from the XRD measurements are shown. Contrary to the XRD data, the Raman spectra do not show any differences with either q^–^ or degraded PA12 content. The present normalization was done to the 1438 cm^–1^ band corresponding to the symmetric stretching −CH2–CH2– vibration.^32^ The materials prepared by cooling at 50 °C·min^–1^ (i.e., with the lowest exposition to high temperatures) were further investigated using TGA (see Figure 6B). Under the inert N_2_ atmosphere, the degradation of all three types of materials does not differ significantly; the mass-loss curves proceed in a single step, with the onset at ∼340 °C and endset at 490 °C. For all three materials, nearly complete gasification occurs, with the solid residue of ∼2–3%. In an oxidizing air atmosphere, the mass-loss mechanism is more complex, with the first step localized between ∼340 and 380 °C (accounting for ∼4% mass loss) and the second, dominant mass loss occurring between 400 and 510 °C, resulting in a solid residue of ∼10–12%. The initial mass-loss step probably indicates the thermo-oxidative attack on methylene groups adjacent to the amide linkages (resulting in the formation of peroxyl radicals and hydroperoxides) and initial hydroperoxide breakdown. In contrast, the dominant mass loss can be attributed to the main-chain scission (β-scission, depolymerization, volatilization reactions affecting C–C and C–N bonds) and final oxidation (combustion of carbonaceous residues).^33^ The prolonged onset (by ∼10 °C) of the major mass-loss step and significant irregularities in the mass-loss curve shape for the nondegraded PA12 material in comparison to materials containing the degraded (already partly thermo-oxidized) PA12 can be explained by a higher number of thermo-oxidation-sensitive sites in the nondegraded polymer structure being responsible for the ongoing formation of peroxyl radicals and hydroperoxides in the first mass-loss step and a higher diversity of subsequent β-scission reactions (producing various compounds, e.g., aldehydes, ketones, carboxyl acids, etc.). The significantly higher nonvolatile content of all of the thermo-oxidatively degraded materials corresponds to the formation of a visually observed foamy char-like residuum (as a product of the oxidizing decomposition).

**Figure 6:**
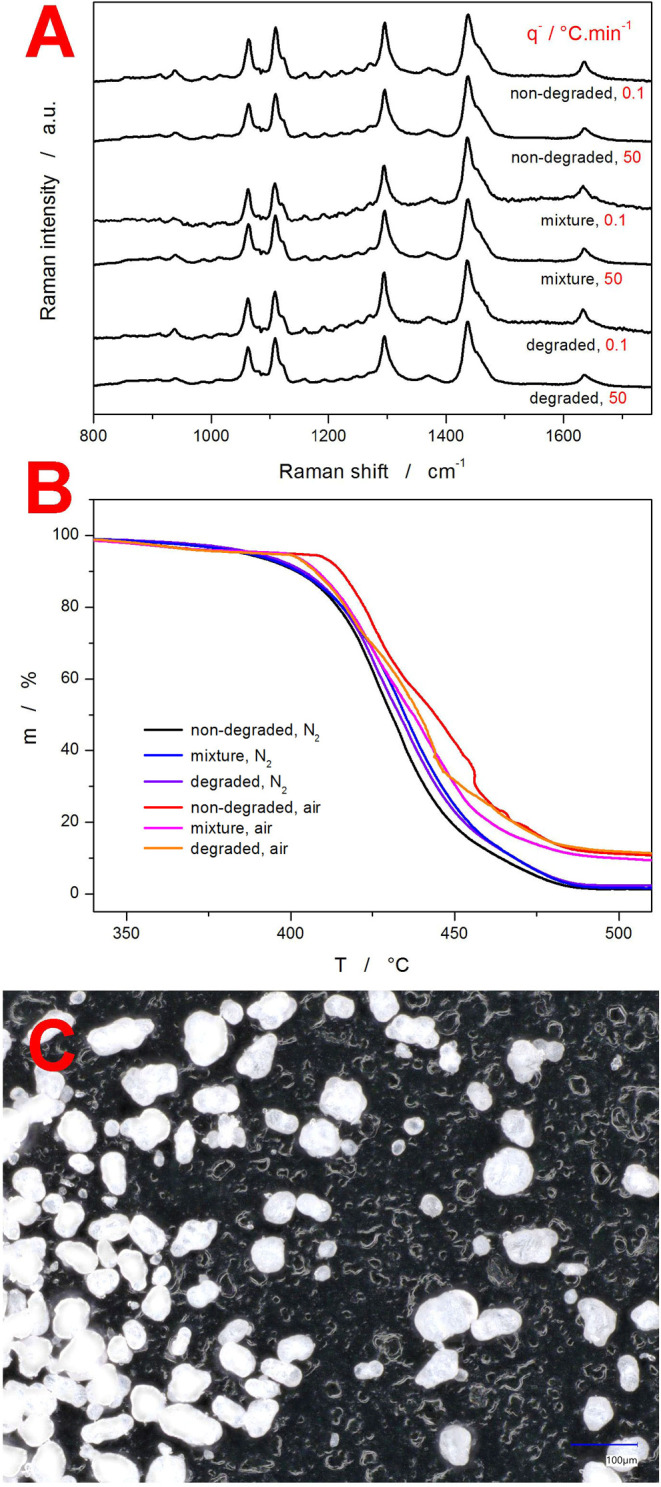
(A) Raman spectra obtained for the set of crystallized PA12 materials made from different powder types at different cooling rates. (B) TGA mass-loss curves obtained under different atmospheres for the crystallized PA12 materials made from different powder types at q^–^ = 50 °C·min^–1^. (C) Example micrograph of the mixture of the nondegraded and degraded PA12 powders.

Furthermore, the morphology of the samples prepared for the XRD and Raman measurements was also investigated by means of optical microscopy. The size and shape of the original powders are represented by Figure 6C (taken for the mixture of the two PA12 powders). Since the shape analyses evaluating the impact of the PA12 powder reuse are already reported in great detail in^15,17^ , we refer the reader to this literature for the details. The micrographs taken for the different PA12 materials after the melting and cooling at different q^–^ are shown in Figure 7. The supramolecular structure formed by cooling the melts at 50 °C·min^–1^ (Figure 7A–E) looks quite similar, with well-developed lamellae radiating from the center of the spherulitic crystals, with the average diameter of the spherulites being ∼20–40 μm. On the contrary, the spherulitic crystallites formed at high temperature during the 0.1 °C·min^–1^ cooling (Figure 7B–F) have less regular shape and less pronounced radially ordered lamellae, which indicates a slow, irregular growth dominating in the energetically favorable directions (rather than radiating strictly outward from the center). These spherulites are significantly larger compared to those formed at lower temperatures: ∼40–60 μm for the nondegraded and degraded materials and ∼70–120 μm for the PA12 mixture. This increase in the spherulite diameter testifies to the growth proceeding through much larger amorphous areas as a consequence of the much lower nucleation density within the melted mixed PA12 material. This further corroborates the above-listed interpretations of the DSC data.

**Figure 7:**
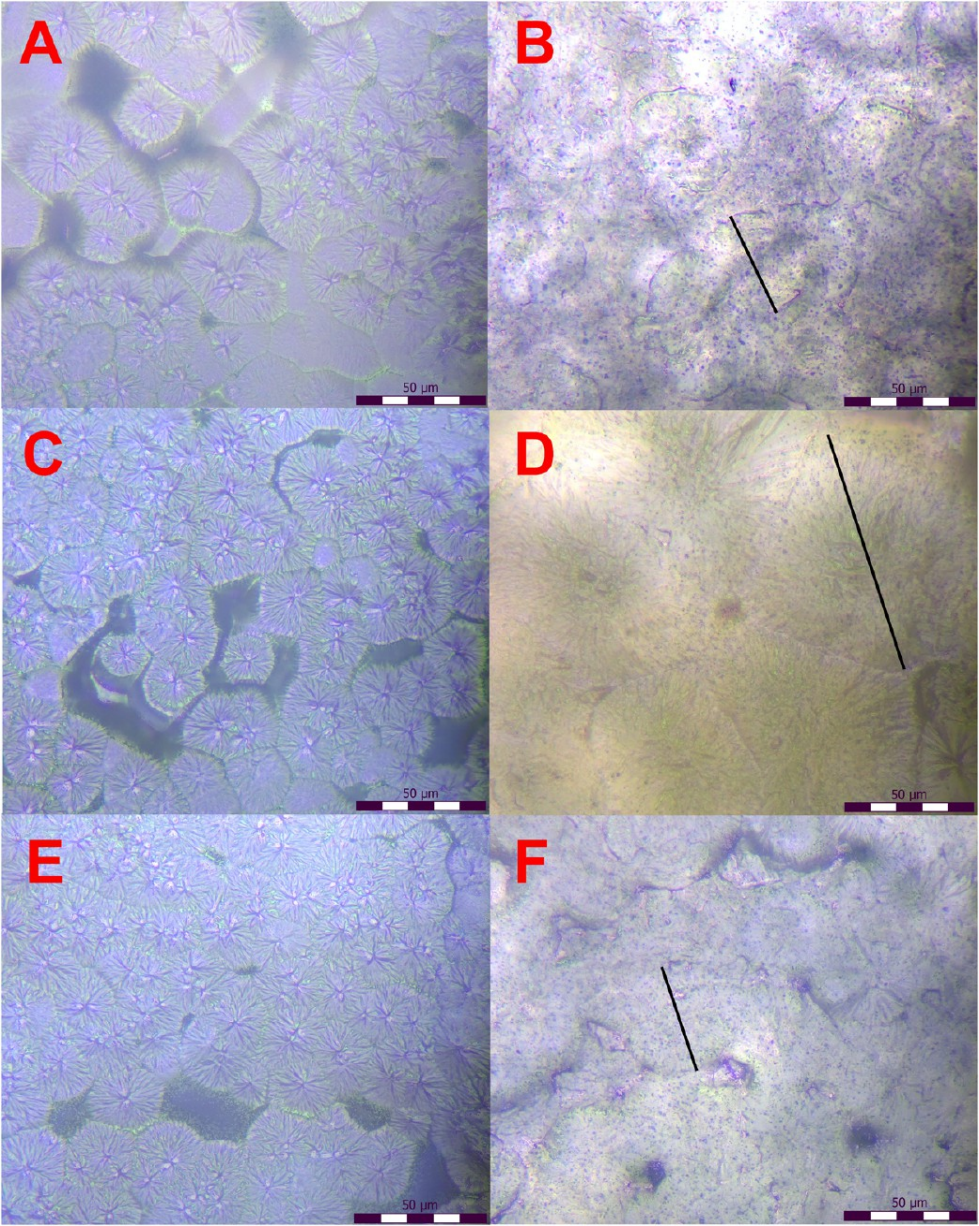
Optical micrographs obtained for the melted PA12 materials made from (A) nondegraded powder cooled at 50 °C·min^–1^; (B) nondegraded powder cooled at 0.1 °C·min^–1^; (C) mixture of powders cooled at 50 °C·min^–1^; (D) mixture of powders cooled at 0.1 °C·min^–1^; (E) degraded powder cooled at 50 °C·min^–1^; (F) degraded powder cooled at 0.1 °C·min^–1^. Parts B, D, and F contain a line indicating the diameter of a typical crystallite.

The impact of the SLS-degraded powder content in PA12 materials on their mechanical properties was explored by means of the frequency and temperature-resolved DMA (note that the standard mechanical tests reported in^16,17^ showed a decrease of the tensile and bending strengths by ∼10–20% for the degraded PA12 powder). A full set of the dynamic DMA data obtained in tensile configuration for the strip prepared from the nondegraded PA12 is shown in Figure 8A, displaying the storage modulus *E*’, loss modulus *E*’’, and loss factor tanδ (defined as *E*’’/*E*’); the curves shift to higher temperature with increasing frequency *f*. In Figure 8B, the comparison of the corresponding measurements (with *f* = 1 Hz) is shown for the strips prepared from all three investigated types of PA12 powders; Figure 8C then shows the determination of the activation energy of structural relaxation *E* obtained from the DMA data according to:^34^

2 $$\ln\left(f\right)=-\frac{E}{RT_{\tan\delta }}+\ln\left(\frac{1}{2\pi \tau_{0}}\right)$$

where *T*_tanδ_ is the temperature corresponding to the maximum of the tanδ peak, and τ_0_ is the pre-exponential factor (for the Arrhenian kinetics usually interpreted as the microscopic attempt time, i.e., the period between the attempts for molecular rearrangement). The comparison shown in Figure 8B demonstrates the complexity associated with the SLS-degradation-induced interactions between the crystalline and amorphous supramolecular structures. Below *T*_g_, the material made of SLS-degraded powder exhibits the increase of stiffness/elasticity (*E*’), a decrease of toughness/plasticity (tanδ), and an increase of *T*_g_ (by ∼3 °C), while the material prepared from the mixture of the two powders shows the opposite: a decrease of *E*’, an increase of tanδ, and a decrease of *T*_g_ (by ∼2 °C). It needs to be noted that the changes in tanδ are only incremental, with all three materials still being highly elastic. Above *T*_g_, the differences between the three materials largely cease. The practical implications of these findings will be introduced in the concluding section; here, we only want to draw attention to the nonmonotonous trends associated with the transition from sole nondegraded to pure degraded raw material (the mixture again shows significantly different properties compared to the arithmetic mean of the properties of the two pure components). The nonmonotonicity is well apparent also in the case of the activation energies of structural relaxation, which imply that the SLS-degradation of the raw PA12 powder largely increases the energetic barriers for the relaxation processes of the resulting material (most probably due to higher molar mass caused by the postcondensation), but the mixture of the powders provides after the melting a material of even more docile molecular structure (most probably due to the significantly different crystalline morphology, see Figure 7D) than the material made of the nondegraded raw powder. It is worth noting that the τ_0_ values determined for the nondegraded, mixed, and degraded powder-based materials were ∼10^–55^, 10^–40^, and 10^–112^, respectively. The much lower values than would be expected for the simple thermally activated molecular processes/reactions (pre-exponential factors in the range of 10^–12^–10^–15^ s) indicate the strong cooperativity of the relaxation motions.^35,36^

**Figure 8:**
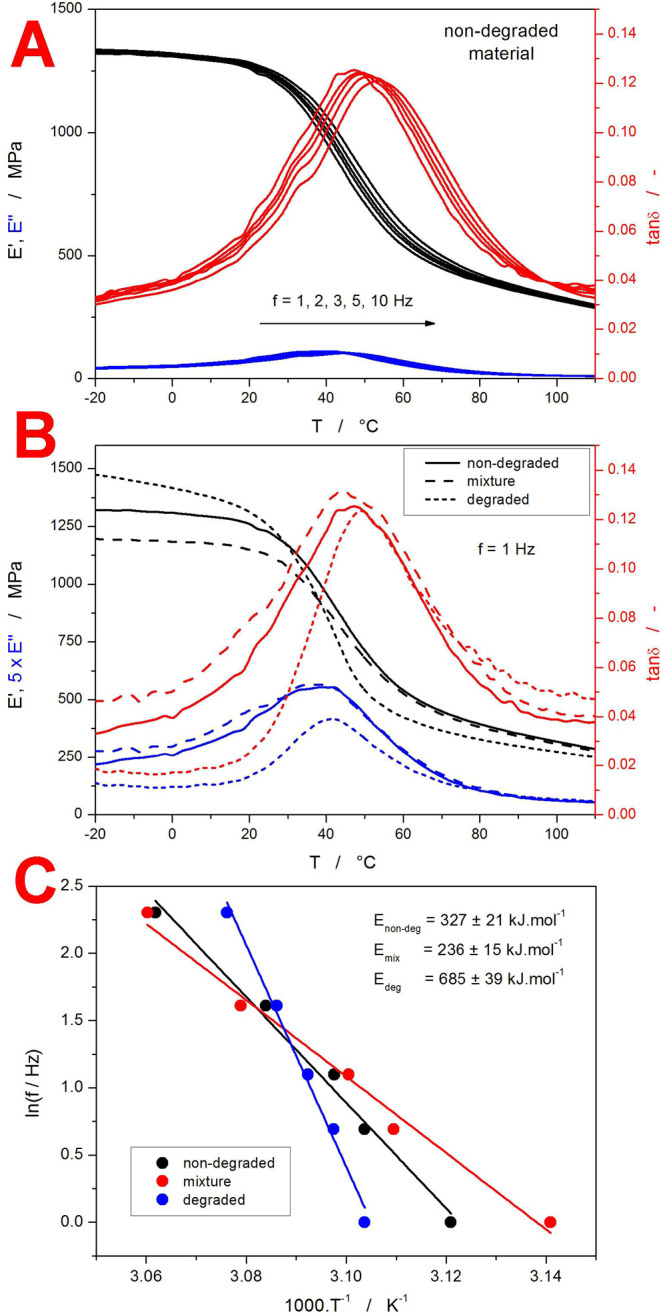
(A) Example of the frequency-dependent DMA data obtained for the PA12 strip compression-molded from the nondegraded powder. (B) Comparison of the DMA data obtained at *f* = 1 Hz for the three types of PA12 materials (differing in the powder type). (C) Evaluation of the activation energy of structural relaxation E according to eq 2 for the three types of PA12 materials.

### 3.2 Advanced Kinetic Description of Macroscopic Crystallization

The macroscopic crystallization kinetics (monitored, e.g., by DSC) in polymeric materials is traditionally described in terms of the Avrami-Hoffman–Lauritzen (AHL) model, combining the idea of the “extended volume” (introduced and tested by Johnson, Mehl, Avrami, and Kolmogorov^37–41^ ) with the Hoffman–Lauritzen crystal growth theory^42^. Mathematically, the description of the DSC data is based on the standard solid-state kinetic equation:^43^

3 $$\frac{d\alpha }{dt}=K\left(T\right)\cdot f\left(\alpha \right)$$

where *K*(*T*) is the Hoffman–Lauritzen expression for the rate constant

4 $$K\left(T\right)=A\cdot \exp\left(-\frac{U^{*}}{R\left(T-T_{\infty }\right)}\right)\exp\left(-\frac{K_{G}}{T\Delta \mathrm{Tf}}\right)$$

where *A* is the pre-exponential factor (essentially translating the manifestation of the exponential dependences to the macroscopic level), U* is the activation energy for the segmental movements of the polymer chains (considered to be 6300 J·mol^–1^ for the majority of polymeric materials^42^), *R* is the universal gas constant, *T*_∞_ is the temperature under which all motions associated with viscous flow are supposed to be significantly higher than the given experimental time scale for the crystal growth (customarily T_∞_ = *T*_g_ – 30 °C, where *T*_g_ is the glass transition temperature), Δ*T* is the undercooling (defined as Δ*T* = *T*_m_^eq^ – *T*, where *T*_m_^eq^ is the equilibrium melting temperature), and *f* is the correction factor defined as *f* = 2*T*/(*T*_m_^eq^ + *T*), and *K*_G_ is the kinetic parameter associated with nucleation, defined as

5 $$K_{G}=\frac{n_{n}b\sigma_{l}\sigma_{f}T_{m}^{\mathrm{eq}}}{\Delta H_{m}^{V}k_{B}}$$

where *n*_n_ is the constant associated with the nucleation regime, *b* is the thickness of a lamellar monolayer, σ_l_ and σ_f_ are the lateral/lamellae and chain-fold free surface energies, respectively, *k*_B_ is the Boltzmann constant, and Δ*H*_m_ *^V^* is the volumetric equilibrium melting enthalpy.

The kinetic model function *f­(*α) in eq 3 is then expressed by the Avrami equation

6 $$f\left(\alpha \right)=m\left(1-\alpha \right){\left[-\ln\left(1-\alpha \right)\right]}^{1-\left(1/m\right)}$$

where *m* is the kinetic exponent related to the nucleation conditions and dimensionality of the forming crystals. Despite being preferred due to its physicochemical interpretability, the AHL model often does not describe the experimental data accurately, which makes the benefit of its interpretability meaningless. For this reason, an MCHL (M-catalytic Hoffman–Lauritzen) kinetic model with maximum flexibility was recently introduced,^18^ where the Hoffman–Lauritzen rate constant utilizes temperature-dependent *K*_g_ and *A* parameters, and the semiempirical Šesták-Berggren equation^43^ is used as the kinetic model

7 $$f\left(\alpha \right)=\alpha^{M\left(T\right)}{\left(1-\alpha \right)}^{N\left(T\right)}$$

where *M* and *N* are the temperature-dependent kinetic exponents characterizing the autocatalytic (*M*) and reaction rate (*N*) components of the kinetic behavior. With the recently introduced^44^ physicochemical interpretation framework of eq 7 (going even beyond the standard nucleation–growth description represented by the AHL model), its main drawback vanished, and the MCHL model can now be considered a truly universal framework for the description of polymer crystallization.

The kinetic description of the polymer crystallization data needs to start with the determination of the characteristic temperatures. For the present PA12 materials, *T*_∞_ was set to 30 °C (well below the calorimetric as well as mechanical *T*_g_), and *T*_m_^eq^ was determined in accordance with the Hoffman–Weeks lamellar thickening method,^45^ where the experimental dependence between *T*_m_ and *T*_c_ is extrapolated toward the *T*_m_ = *T*_c_ state. The Hoffman–Weeks plot constructed for the present nondegraded PA12 data is shown in Figure 9A. The *T*_c_ values were taken either from the isothermal DSC measurements (using the annealing temperatures) or from the nonisothermal measurements (*T*_c_ evaluated as the temperature corresponding to the maximum of the crystallization peak). On the other hand, the melting peak exhibited a complex shape for the majority of experimental conditions, consisting of two overlapping peaks corresponding to the γ and α polymorphic phases. Whereas the melting prepeak shows a characteristic increase of *T*_m_ with increasing *T*_c_, the *T*_m_ value corresponding to the main melting peak stagnates at ∼177 °C. This indicates that the γ phase (represented by the melting prepeak) forms only during the cooling, while a significant portion of the α phase (represented by the main melting peak) forms during the recrystallization from the melt (after the γ phase melts within the prepeak). For this reason, the *T*_m_^eq^ = 187.3 °C determined for the γ phase of the nondegraded PA12 will be used in the further calculations; the value is close to the literature values (192–194 °C^46,47^ ).

**Figure 9:**
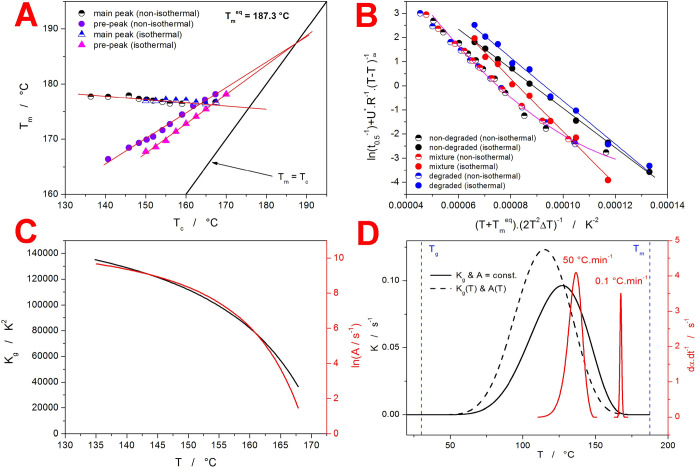
(A) Hoffman–Weeks plot for the nondegraded PA12 material. (B) Determination of *K*_g_ according to eq 8 from the nonisothermal and isothermal DSC data obtained for the three types of the PA12 materials. (C) Temperature-dependent *K*_g_(*T*) and ln *A*(*T*) values determined from the polynomial fit of the nonisothermal data shown in part B. (D) *K*(*T*) dependences (left axis) and crystallization data (right axis) obtained for the nondegraded PA12 material.

Whereas the majority of the MCHL model parameters are best determined via nonlinear optimization (curve-fitting of the experimental data by the combination of eqs 3, 4, and 7), it is beneficial to reduce the number of variables by independent determination of the *K*_g_(*T*) temperature dependence and thus eliminate the mutual compensation between *K*_g_ and pre-exponential factor *A*. The corresponding methodology is based on the linearization approach utilizing the *t*_0.5_ values (time for which α = 0.5)

8 $$\ln\left(1/t_{0.5}\right)+\frac{U}{R\left(T_{c}-T_{\infty }\right)}=\ln A-\frac{K_{G}}{T_{0.5}\Delta \mathrm{Tf}}$$

where *T*_0.5_ is the temperature corresponding to t_0.5_. This linearization is for both types of experimental data (isothermal and nonisothermal) and all three PA12 materials depicted in Figure 9B. The isothermal crystallization data show good linearity, providing the following *K*_G_ values: 81,800 ± 900 K^2^ for nondegraded material, 89,500 ± 3200 K^2^ for the degraded material, and 109,400 ± 3900 K^2^ for the mixture. Since *K*_g_ is related to the energetic barrier for the secondary nucleation, this finding immediately points to the hindered nucleation as the fundamental reason for the significant crystallization slowdown observed for the remelted mixture of the two PA12 powders (qualitatively described in Section 3.1). Contrary to the isothermal data shown in Figure 9B, all nonisothermal dependences exhibit pronounced curvatures (with all the data practically overlapping). By fitting the dependences with a second-order polynomial function and deriving the corresponding equation, a temperature dependence of *K*_G_ and ln­(*A*/s^–1^) can be obtained, as depicted in Figure 9C (for the purpose of the present study, a sole fit was performed for all nonisothermal data in Figure 9B). The obtained *K*_g_ values are generally conformable to the literature data for the PA12 material: the value of 113 kK^2^ evaluated from the nonisothermal data (q^–^ = 0.2–10 °C·min^–1^) was reported in^48^; values of 80 and 39 kK^2^ were reported for the low-T and high-T crystal growth regimes monitored^49^ microscopically under isothermal conditions; the value of 136 kK^2^ was obtained from the isothermal DSC experiments in^46^.^46,46,46,46,46^

With the knowledge of *T*_m_^eq^, *T*_∞_, *K*_G_, and ln *A*, the temperature dependence of the crystallization rate constant *K*(*T*) can be modeled according to eq 4. The *K*(*T*) constants simulated for both sets of the *K*_G_ and ln *A* parameters (two constants from the isothermal data versus the temperature dependences depicted in Figure 9C) are compared in Figure 9D with the nonisothermal crystallization data obtained for the nondegraded PA12 at the two extreme cooling rates (0.1 and 50 °C·min^–1^). The main purpose of this comparison is the evaluation of the mutual positions of the crystallization peak and the point of inflection on the onset side of the *K*(*T*) dependence. It was shown recently^27^ that for the Avrami crystallization peaks positioned above the *K*(*T*) inflection point (closer to the *K*(*T*) maximum), the typical asymmetry of the peak (associated with the Avrami kinetics) largely changes into skewing toward lower α. Despite the study^27^ deals with the crystallization data obtained during heating of the amorphous phase, the same conclusion also holds for the crystal growth occurring during cooling of the melt (such as in the present case of PA12 polymer). Compared to the data obtained at 0.1 °C·min^–1^, the crystallization peak obtained at 50 °C·min^–1^ is clearly significantly more skewed toward lower α (higher T). This indicates that the crystallization at 50 °C·min^–1^ should already proceed above the *K*(*T*) inflection point, which, in consequence, points to the *K*(*T*) calculated on the basis of constant *K*_G_ and *A*. Considering the very good linearity (in agreement with the theoretical background) of the isothermal dependences in Figure 9B, the temperature-independent values of *K*_G_ were preferred in the nonlinear optimizations.

With the temperature dependence of the rate constant *K*(*T*) being determined, the experimental data can be modeled based on the choice of the appropriate kinetic model f­(α). The Levenberg–Marquardt algorithm was employed to minimize the sum of squared residue in accordance with the single-curve multivariate kinetic analysis (sc-MKA)^50^ modified for the Hoffman–Lauritzen kinetics (having *K*_g_ fixed during the optimization)

9 $$\mathrm{SSR}=\sum_{{\mathrm{First}}_{j}}^{{\mathrm{Last}}_{j}}{\left({Y\exp}_{j,k}-{Y\mathrm{cal}}_{j,k}\right)}^{2}$$

where SSR is the sum of squared residue, *j* is the index of the given measurement, First_*j*_ is the index of the first point of the given curve, Last_*j*_ is the index of the last point of the given curve, *Y*exp_*j,k*_ is the experimental value of the point *k* of curve *j*, and Ycal_*j,k*_ is the calculated value of the point *k* of curve *j* (calculated in accordance with eqs 3, 4 and 6 or 7).

The difference between the AHL and MCHL models is demonstrated in Figure 10A; the fit was performed for the crystallization peak obtained at 0.1 °C·min^–1^ for the nondegraded PA12. The following curve-fitting metrics represent the data: fit by the AHL model is characterized by the correlation coefficient *r* = 0.9868 and a sum of squared residue SSR = 0.0031, while the fit by the MCHL model is characterized by the correlation coefficient *r* = 0.9992 and a sum of squared residue SSR = 0.0002. Clearly, the AHL model cannot fit the experimental data accurately; the identical manifestation of the “Avrami” and “Avrami­(T)” fits also demonstrates that the temperature-dependent *K*_g_(*T*) and ln *A*(*T*) compensate for each other but cannot change the characteristic Avrami asymmetry. Similar or even larger inaccuracies were also reported in all other literature studies utilizing the AHL concept for the description of the PA12 crystallization data.^46–49^ Correspondingly, it should be mentioned that the values of the Avrami kinetic exponent reported based on such inaccurate data fits lack meaning (and thus will not be discussed in the present paper). The inability of the AHL model to describe the DSC peaks skewed to low α values naturally favors the flexible (and now also physically meaningful^44^) MCHL model. The quality of the MCHL fits is for the full set of the nondegraded PA12 crystallization peaks demonstrated in Figure 10B,C. Similarly, good correlation was also obtained for the other data sets (measured nonisothermally and isothermally for all three PA12 materials) (see Section S3).

**Figure 10:**
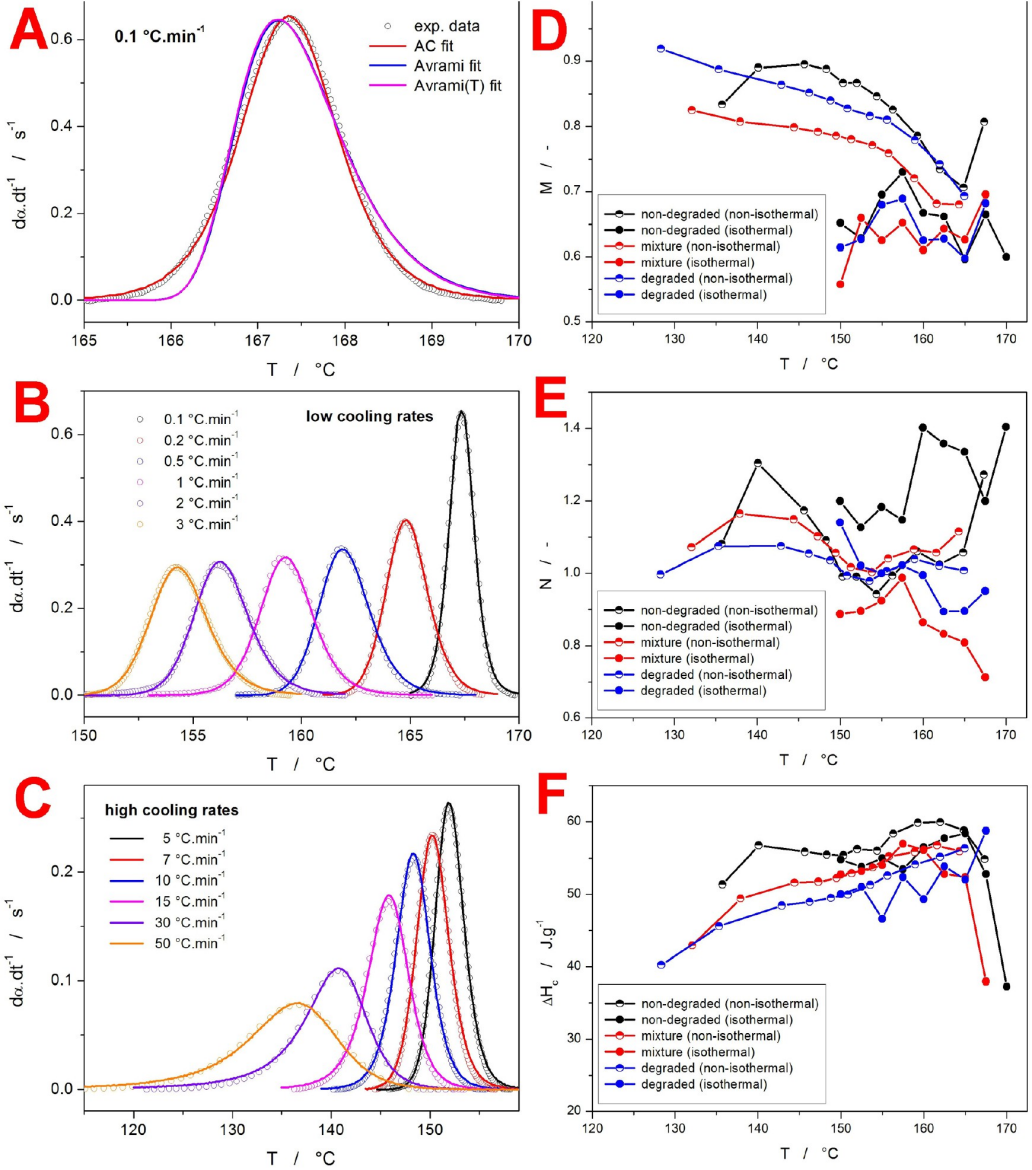
(A) Crystallization peak obtained at q^–^ = 0.1 °C·min^–1^ for the nondegraded PA12 material fit by the AHL, AHL­(T), and MCHL models. (B, C) Crystallization peaks obtained for the nondegraded PA12 fit by the MCHL model. (D–F) Kinetic parameters determined by the sc-MKA method for the crystallization data of all three PA12 materials (differing in the content of the degraded PA12).

The parameters of the sc-MKA fits (kinetic exponents *M* and *N*, and crystallization enthalpies) are displayed in Figure 10D–F. Starting with the autocatalytic exponent *M*, the isothermal data show values *M* ≈ 0.6–0.7, while the nonisothermal data exhibited *M* ≈ 0.7–0.9. Since this parameter primarily testifies about the degree of the autocatalytic behavior (initiated, e.g., by the presence/formation of the pre-existing/secondary nuclei, by impurities, or phase-separated domains), the generally low *M* values associated with the isothermal data (+ nonisothermal data for the PA12 mixture) can be associated with the low number of initial nuclei and higher energetic barrier for the crystallization process.^44^ The values of parameter *N* vary mostly between 0.8 and 1.4, where *N* > 1 indicates a deviation from the nucleation–growth Avrami asymmetry, manifesting through the peak skewing to lower α values (identifiable by the prolonged endset tail of the crystallization peak in the more extreme cases). The physicochemical interpretation of this feature is associated with, e.g., molecular mobility being constrained due to internal stresses arising from the preceding formation of the crystalline phase, broad quality distribution of nucleation sites, or secondary crystallization being initiated from newly formed active sites at an already existing crystalline surface.^44^ Since the highest *N* values are found for the nondegraded PA12 material, it is probably the secondary nucleation that dominates this effect. As the crystallinity and spherulites packing appear to be roughly similar for all three PA12 materials, the only other cause for changing *N* (namely, its decrease in the case of the slowly crystallizing PA12 mixture) may be the lower internal stresses formed as a consequence of low nucleation density (see Figure 7D). Finally, the crystallization enthalpies depicted in Figure 10F show a decrease not only with increasing q^–^ (which may be explained by the combined contributions of the Kirchhoff’s law and of shifting the polymorphic equilibrium) but also with a marked decrease in ΔH_c_ observed for the materials with nondegraded PA12 content at very low q^–^. The latter can be a consequence of the isothermal measurements not being fully finished within the framework of the programmed DSC experiment.

### 3.3 Advanced Kinetic Description of Microscopic Crystallization

Microscopic observation of crystal growth was used as a complementary technique to the DSC measurements to provide further insight into the differences in the crystallization behavior of the investigated PA12 powders. The crystal growth rate was measured isothermally, using the hot-stage polarized microscopy, at annealing temperatures *T*_a_ = 160, 165, 170, and 175 °C, after melting the original powder at either 190 or 230 °C. For each *T*_a_, the micrographs were taken for a series of increasing annealing times *t*_a_, until practically full crystallinity was achieved. An exemplary micrograph obtained for the SLS-degraded PA12 material annealed for 30 min at 170 °C (after being thoroughly melted at 230 °C) is shown in Figure 11A. The measurements of the spherulite size were done using calibrated QuickPhoto software. In particular, the diameter of the 10–30 largest solitary crystallites was taken for each combination of *T*_a_ and *t*_a_ to avoid interference of the continuing nucleation and crystal impingement effects. The micrographs taken at high *t*_a_s, when no solitary (or impingement-unaffected) spherulites were present, were omitted from the evaluation. A data set of average spherulites’ diameters measured for the SLS-degraded PA12 material melted at 230 °C and then crystallized at different *T*_a_s is shown in Figure 11B. These data were consequently used to calculate the crystal growth rate *u*_r_, reflecting the propagation of the growth front (radius of the spherulite) from its center/nucleus. For the determination of *u*_r_, only the linear parts of the d-*t* dependences were used to eliminate the errors originating from the effects of the nucleation induction time (delay between the onset of the isotherm and first occurrence of the nuclei/crystals) and crystals impingement (final slowdown of crystal growth due to the direct contact with other crystal or due to the diffusion/stress barriers arising within the remaining amorphous pockets).

**Figure 11:**
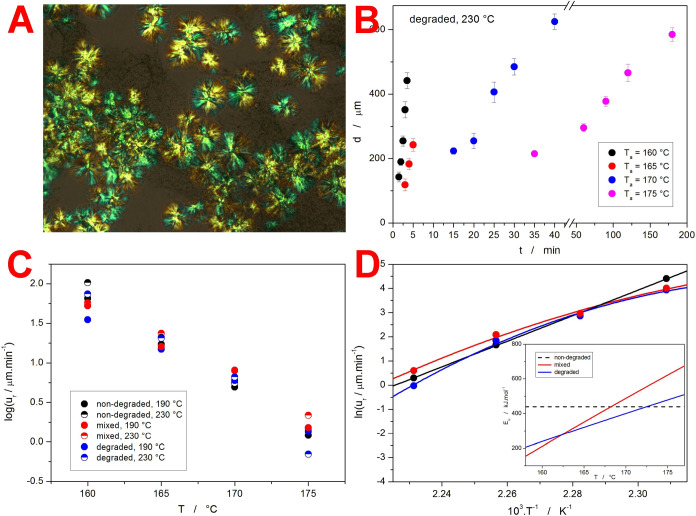
(A) Exemplary polarized micrograph obtained for the degraded PA12 material annealed for 30 min at 170 °C after being melted at 230 °C. The micrograph dimensions are 3580 × 2693 μm. (B) Evolution of the crystallite diameters with annealing time, *t*_a_, obtained at different *T*_a_s for the degraded PA12 material melted at 230 °C. (C) Crystal growth rates determined for the different studied PA12 materials melted at 190 or 230 °C. (D) Evaluation of *E*_u_-*T* dependences for the studied PA12 materials (points were obtained as averages from the 190 and 230 °C data points shown in part C; lines represent either linear or 2nd-order polynomial fits of the data). The inset shows the determined *E*_u_-*T* dependences.

The crystal growth rates determined for the present three PA12 materials are displayed in Figure 11C. Despite certain variability, the growth rates are in absolute values roughly similar (certainly within the same order of magnitude) for all three PA12 materials, regardless of the temperature at which the materials were melted. To calculate the activation energy of crystal growth *E*_u_ according to:^51^

10 $$\frac{d\ln\left(u_{r}\right)}{d\left(1/T\right)}=\frac{E_{u}}{R}$$

the *u*_r_ values obtained for the two melting temperatures (190 and 230 °C) were averaged and fit by either a straight line (nondegraded PA12) or by the second-order polynomial functions (mixed and degraded PA12 materials). The fits and the corresponding *E*_u_-*T* dependences are shown in Figure 11D. Clearly, the SLS-degradation leads during the consequent powder fusion (as done in the present study) to an alteration of the activation energy of the crystal growth process, where at high temperatures, *E*_u_ increases above that of the nondegraded material, while at low temperatures, *E*_u_ significantly decreases. This is a key finding for the interpretation of the XRD data from Figure 5C (as advertised in the corresponding discussion in Section 3.1). Due to the higher activation energy barriers and overall slightly slower crystal growth at high temperatures (see Figure 11D), better lamellae ordering can develop for the very slowly cooled SLS-degraded PA12 material, which will result in more prominent XRD peaks. In contrast, the significantly lower *E*_u_ calculated for the SLS-degraded material at low temperatures (compared to the nondegraded material) leads to fast growth with less regular/ordered lamellae packing and the corresponding low intensity of the XRD peak.

## 4 Conclusions

The present data demonstrate that SLS-degraded (recycled) PA12 powder can be reused in conventional melt processing, but its crystallization and morphological behavior differ significantly from both virgin (nondegraded) powder and simple compositional predictions. The DSC analysis revealed that the mixture of the nondegraded and SLS-degraded powders in the weight ratio of 25/75 crystallizes substantially more slowly at low cooling rates and during isothermal annealing compared to the basic two types of powders. This effect stems from reduced nucleation density caused by the molecular incompatibility between regular nondegraded polymer chains and the higher-molar-mass, structurally irregular chains produced by SLS-induced postcondensation. The blend, therefore, experiences kinetically hindered early stage crystallization and a shift toward γ-phase formation. Structural and microscopic analyses confirmed that the three PA12 materials, although chemically similar, differ strongly in crystalline morphology. XRD revealed higher apparent crystallinity for degraded PA12 at slow cooling, consistent with polarized optical microscopy showing temperature-dependent crystal growth behavior and large spherulites in the powder mixture due to sparse nucleation. Application of the flexible MCHL kinetic model captured these effects accurately and identified elevated *K*_G_ values for the powder mixture, confirming its increased nucleation barrier. Owing to the accurate data description by the MCHL model and to the recently developed interpretational framework of its parameters, the fundamental physicochemical explanation of the responsible nucleation/crystallization mechanisms was obtained, confirming the critical role of secondary nucleation and molecular incompatibility of the nondegraded and highly degraded PA12 powders.

Mechanical testing showed that the degradation and powder blending influence PA12′s storage modulus, damping behavior, and relaxation dynamics in a nonmonotonic manner, reflecting the strong coupling between chain architecture, crystallization pathways, and supramolecular structure. Whereas the ∼10% increase in Young’s modulus observed for the sole degraded PA12 material below 25 °C can be technologically beneficial for certain applications (e.g., electrical housings and brackets, fasteners and snap-fits, vibration-sensitive parts, bushings, and guide rails used at ambient/cold conditions), it has to be borne in mind that above 25 °C, the mechanical stiffness of the degraded material was considerably lower than that of the other two tested PA12 materials. On the other hand, the material formed from the mixture of the two powders exhibited significantly lower Young’s modulus at ambient and subambient temperatures, but above 50 °C, its mechanical properties were practically identical to those of the nondegraded material. Considering the high content of degraded material in the mixture, this may be an interesting alternative for recycling SLS-degraded PA12 powders in applications where PA12 parts are manufactured by melt processing and subsequently subjected to loads at elevated temperatures. These findings clearly demonstrate the value of the detailed studies reporting on the temperature-dependent mechanical properties (oscillation DMA data) in contrast to the standard mechanical tests that do not reveal these possibilities.

Overall, the obtained findings provide a detailed, multiscale understanding of how SLS-induced degradation affects the crystallization and performance of PA12 in melt-processing environments. Most importantly, they show that mixtures of degraded and nondegraded powders behave as unique materials rather than intermediate compositions. This finding enables the formulation of rational processing guidelines: while degraded powder alone remains suitable for reuse in non-SLS melt-processing techniques, its blending with virgin powder requires careful control of cooling conditions to avoid kinetic suppression and undesirable morphological outcomes. The advanced crystallization modeling approach via the MCHL model employed here offers a robust framework for predicting and optimizing such behaviors and can be applied broadly to other thermoplastic polymers subjected to thermal or processing-induced degradation.

## Supplementary Material



## Data Availability

The data are available at the Figshare repository (10.6084/m9.figshare.30815714).

## Notes

Experimental setups, thermo-analytical characteristics of DSC data, nonisothermal and isothermal DSC data fit by the MCHL model (PDF)
