# Supplementary material for: Recycling Potential of SLS-Degraded PA12 Powder for Melt-Based Manufacturing: Insights Resulting from the Advanced Crystallization Modeling
Source: ACS Omega. 2026 Jul 15;11(29):44044–60. doi: 10.1021/acsomega.6c03917 (PMC13425315; doi:10.1021/acsomega.6c03917)
Supplement: Supplementary file 1 [file ao6c03917_si_001.pdf]

Electronic Supplementary Information File

Recycling potential of SLS-Degraded PA12 Powder for Melt-Based Manufacturing: Insights Resulting from the Advanced Crystallization Modeling

Roman Svoboda[1*], Jakub Vlachynský[2], David Jaška[2], Jana Machotová[3], Jana Navrátilová[2]

[1]*Department of Physical Chemistry, Faculty of Chemical Technology, University of Pardubice, Studentská 573, 532 10 Pardubice, Czech Republic.*

[2]*Department of Polymer Engineering, Faculty of Technology, Tomas Bata University in Zlín, Vavrečkova 5669, 760 01 Zlín, Czech Republic*

[3]*Institute of Chemistry and Technology of Macromolecular Materials, Faculty of Chemical Technology, University of Pardubice, Studentská 573, 532 10 Pardubice, Czech Republic.*

Section S1: Experimental setups used in the present study

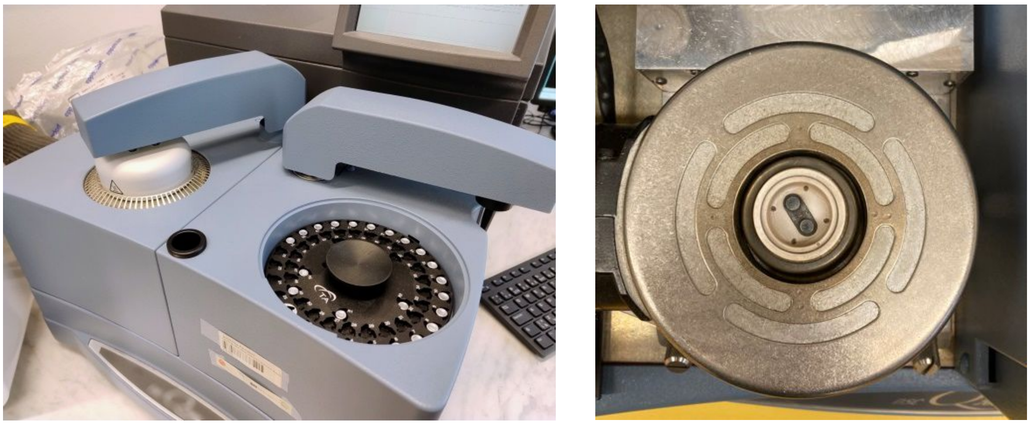

Fig. S1: DSC instrument (left), open DSC cell (right).

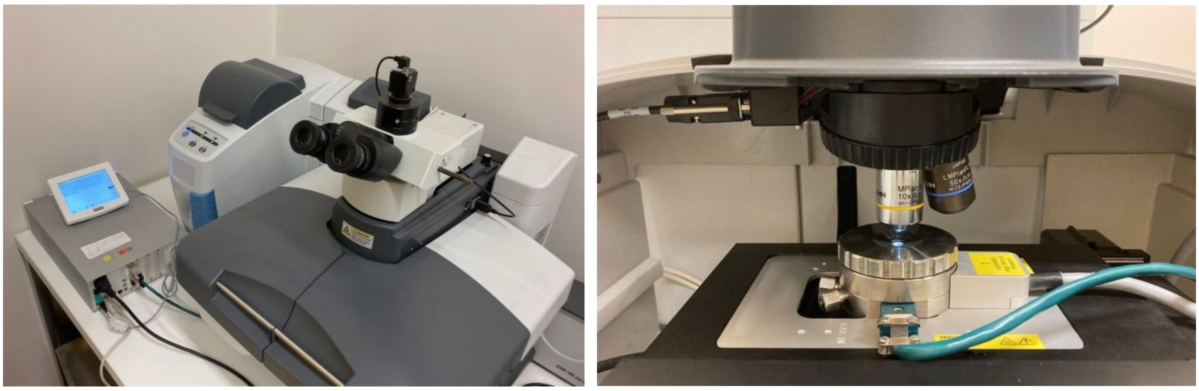

Fig. S2: Raman microscope (left), hot-stage cell inside the Raman microscope (right).

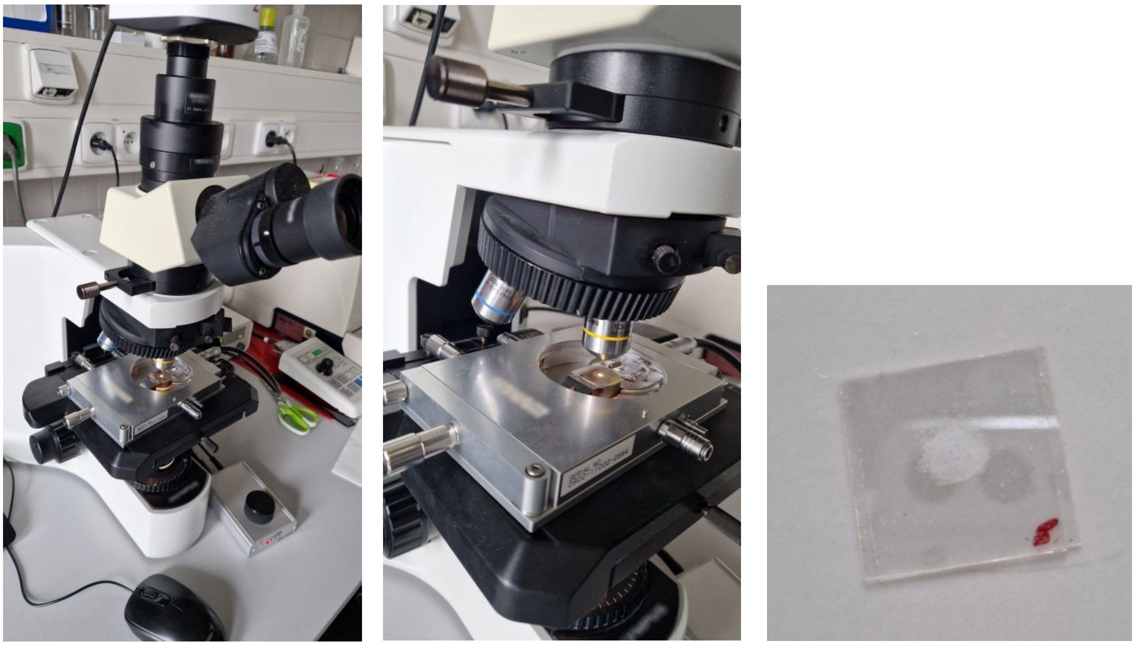

Fig. S3: Optical microscope (left), hot-stage (middle), PA12 powder between microscopic slides (left).

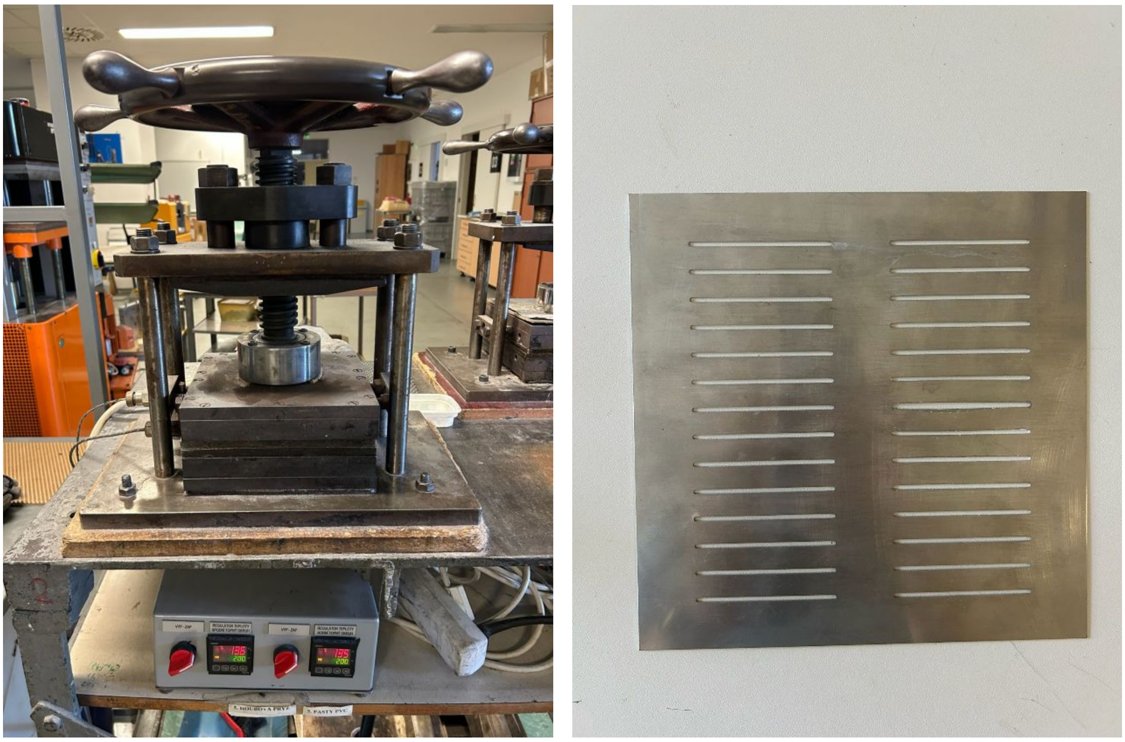

Fig. S4: Preparation of the strip samples for DMA – heated press (left), form filled with compression molded samples (right).

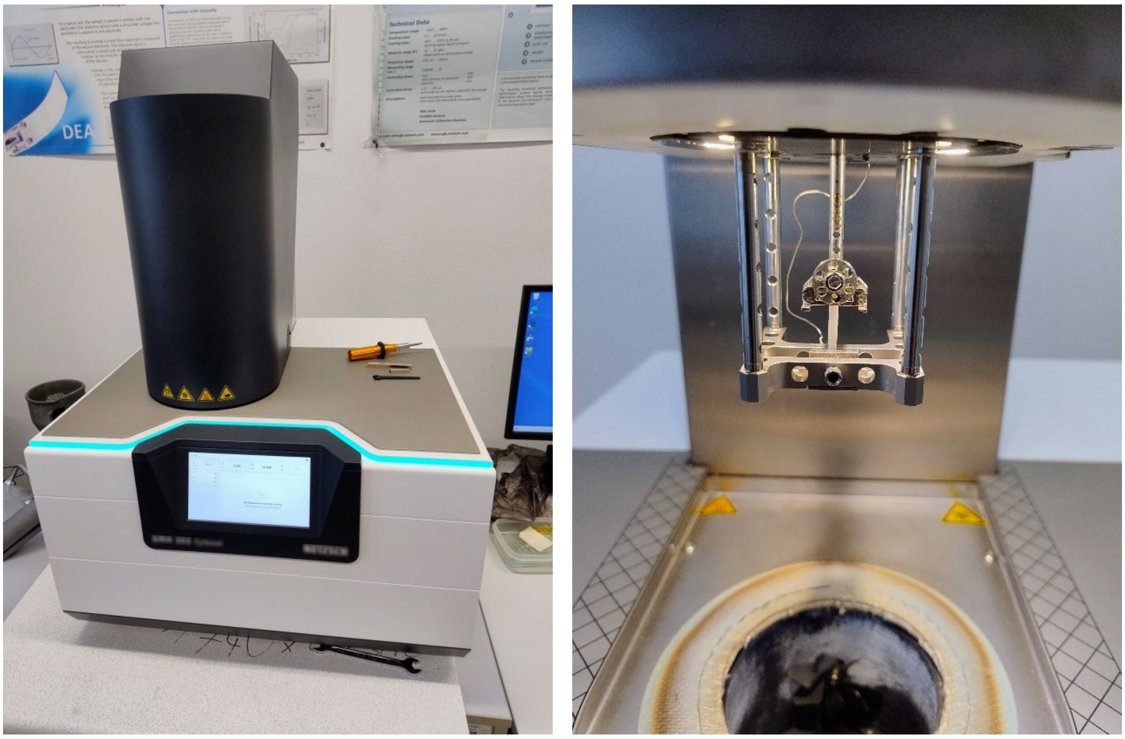

Fig. S5: DMA instrument (left), PA12 strip sample clamped in the tensile setup (right).

Section S2: Thermo-analytical characteristics of DSC data.

Tables S1-S3: Thermo-analytical characteristics of the raw non-isothermal DSC cycles (such as shown, e.g., in Fig. 2C) obtained for the PA12 materials. T_c^{ons}, T_c^p, and H_c correspond to the onset temperature, peak temperature and enthalpy determined for the crystallization peak measured at q^- during the cooling part of the cycle. Subscripts "m1" and "m2" indicate the onset and peak temperatures obtained for the first and second melting peaks. The melting enthalpy was determined in two ways: H_m is integrated between the onset and endset of the main melting peak/double-peak, H_m^* is integrated between the onset of the RAF signal and the endset of the main melting peak/double-peak.

Table S1: Data for the non-degraded PA12 material.

q^-	T_c^{ons}	T_c^p	H_c	T_{m1}^{ons}	T_{m1}^p	T_{m2}^{ons}	T_{m2}^p	H_m	H_m^*
°C·min^{-1}	°C	°C	J·g^{-1}	°C	°C	°C	°C	J·g^{-1}	J·g^{-1}
0.2	166.8	164.76	60.76	173.02	177.05	173.02	177.05	59.85	59.85
0.5	164.32	161.81	58.32	172.07	176	172.07	176	59.36	59.36
1	161.85	159.05	56.83	170.56	175.11	170.56	175.64	59.13	59.13
2	158.97	156.02	56.93	168.76	172.73	169.04	176.47	46.72	57.57
3	157.11	154.18	56.03	168.14	171.63	17068	176.73	45.46	56.75
5	154.94	151.87	55.12	167.04	170.36	172.28	176.98	44.33	55.86
7	153.51	150.25	54.71	166.88	169.73	172.36	177.18	43.18	55.87
10	151.88	148.36	55.1	166.4	169.25	172.69	177.33	42.99	54.65
15	149.88	145.88	55.45	166.25	168.46	172.49	177.95	42.69	54.68
30	146.41	140.75	56.56	165.93	166.72	172.3	177.67	44.23	58.02
50	144.83	136.39	55.58			172	177.73	45.66	54.82

Table S2: Data for the mixed PA12 material.

q^-	T_c^{ons}	T_c^p	H_c	T_{m1}^{ons}	T_{m1}^p	T_{m2}^{ons}	T_{m2}^p	H_m	H_m^*
°C·min^{-1}	°C	°C	J·g^{-1}	°C	°C	°C	°C	J·g^{-1}	J·g^{-1}
0.2	166.1	164.24	59.26	172.88	176.5	172.88	176.5	58.61	58.61
0.5	163.78	161.42	57.94	172.28	175.52	172.28	175.52	45.66	56.78
1	161.36	158.63	56.67	170.69	173.88	170.69	174.47	46.04	56.01
2	158.52	155.53	57.08	168.77	171.97	168.83	176.7	43.71	56.22
3	156.73	153.62	56.04	167.77	171.15	171.2	176.47	42.28	54.58
5	154.59	151.2	53.58	167	170.25	171.85	176.93	40.92	52.87
7	153.12	149.45	51.84	166.43	169.68	171.88	176.97	40.14	52.07
10	151.42	147.4	51.55	165.86	169.29	171.94	177	39.86	52.49
15	149.3	144.64	51.07	165.09	168.16	171.88	176.69	39.54	51.25
30	144.91	138.38	50.33	164.44	166.49	171.29	176.53	38.54	50.91
50	140.81	132.57	48.87			170.52	176.4	39.26	51.24

Table S3: Data for the degraded PA12 material.

q^-	T_c^{ons}	T_c^p	H_c	T_{m1}^{ons}	T_{m1}^p	T_{m2}^{ons}	T_{m2}^p	H_m	H_m^*
°C·min^{-1}	°C	°C	J·g^{-1}	°C	°C	°C	°C	J·g^{-1}	J·g^{-1}
0.2	166.77	164.8	55.22	173.51	177.39	173.51	177.39	56.81	56.81
0.5	164.11	161.78	57.19	172.67	176.11	172.67	176.11	57.1	57.1
1	161.42	158.7	55.84	170.87	174.83	170.87	175.62	43.68	55.04
2	158.43	155.32	54.98	168.81	173.05	168.81	177.25	41.49	54.3
3	156.63	153.29	54.76	168.14	172.26	171.56	177.12	39.58	54.63
5	154.33	150.74	54.05	167.67	171.31	171.91	177.37	37.75	55.06
7	152.6	148.8	51.86	167.04	170.68	172.44	177.44	37.24	54.91
10	150.54	146.33	49.41	166.25	169.88	172.09	177.55	37.32	52.85
15	147.99	143.01	48.66	165.45	168.62	172.12	177.23	36.41	54.65
30	142.48	135.68	47.31	164.66	167.04	171.28	176.69	35.25	51.72
50	137.41	128.83	46.71			170.46	176.33	36.03	49.74

Tables S4-S6: Thermo-analytical characteristics of the raw non-isothermal DSC measurements following the isothermal annealings at different T_as obtained for the PA12 materials. T_{RAF}^{ons}, T_{RAF}^p, and H_{RAF} correspond to the onset temperature, peak temperature and enthalpy determined for the endothermic signal associated with the RAF phase formed during the isothermal crystallization. Subscripts "m1" and "m2" indicate the onset and peak temperatures obtained for the first and second melting peaks. The melting enthalpy was determined in two ways: H_m is integrated between the onset and endset of the main melting peak/double-peak, H_m^* is integrated between the onset of the RAF signal and the endset of the main melting peak/double-peak.

Table S4: Data for the non-degraded material.

T_a	T_{RAF}^{ons}	T_{RAF}^p	H_{RAF}	T_{m1}^{ons}	T_{m1}^p	T_{m2}^{ons}	T_{m2}^p	H_m	H_m^*
°C	°C	°C	J·g^{-1}	°C	°C	°C	°C	J·g^{-1}	J·g^{-1}
150	151.14	152.96	1.06	166.3	167.84	172.31	176.95	44.84	53.87
152.5	153.53	155.26	1.03	166.99	168.65	172.6	176.97	43.72	53.66
155	155.97	157.53	0.75	167.99	169.53	172.86	177.12	44.8	53.46
157.5	158.5	159.8	0.63	168.93	171.13	172.81	177.05	45.3	53.31
160	161.19	162.26	1.04	170.44	172.81	173.42	177.01	54.89	54.89
162.5	163.69	164.71	0.79	172.02	173.94	172.02	173.94	54.4	54.4
165	166.73	167.75	0.62	173.49	175.42	173.49	175.42	56.09	56.09
167.5	169.67	170.49		174.32	176.65	174.32	176.65	55.98	55.98
170				173.97	178.15	173.97	178.15	56.49	56.49

Table S5: Data for the mixed material.

T_a	T_{RAF}^{ons}	T_{RAF}^{p}	H_{RAF}	T_{m1}^{ons}	T_{m1}^{p}	T_{m2}^{ons}	T_{m2}^{p}	H_m	H_m^*
°C	°C	°C	$J{\cdot}g^{-1}$	°C	°C	°C	°C	$J{\cdot}g^{-1}$	$J{\cdot}g^{-1}$
150	151.04	152.75	1.05	165.48	166.47	171.08	176.48	46.2	53.29
152.5	153.39	154.9	0.84	165.45	166.94	170.98	176	44.44	52.83
155	155.81	157.17	0.7	167.1	168.7	172.3	176.77	43.63	53.26
157.5	158.53	159.58	0.94	167.69	169.72	172.82	176.16	45.41	54.15
160	160.95	161.95	0.89	169.73	171.86	172.44	176.75	45.46	54.19
162.5	164.02	164.83	0.56	170.56	172.98	170.56	175.17	45.3	54.6
165	166.22	167.04	0.51	172.6	174.92	172.6	174.92	54.09	54.09
167.5				173.36	175.65	173.36	175.65	51.85	51.85
170				170.92	177.33	170.92	177.33	55.02	55.02

Table S6: Data for the degraded material.

T_a	T_{RAF}^{ons}	T_{RAF}^{p}	H_{RAF}	T_{m1}^{ons}	T_{m1}^{p}	T_{m2}^{ons}	T_{m2}^{p}	H_m	H_m^*
°C	°C	°C	$J{\cdot}g^{-1}$	°C	°C	°C	°C	$J{\cdot}g^{-1}$	$J{\cdot}g^{-1}$
150	151.22	153.04	1.08	166.68	167.82	172.47	177.18	42.25	49.41
152.5	153.57	155.33	1.02	167.27	168.67	173	177.39	42.28	50.38
155	156.02	157.66	0.76	168.28	169.81	173.12	177.37	41.52	48.89
157.5	158.53	159.98	0.63	169.11	171.12	173.77	177.55	44.83	51.14
160	161.31	162.38	1.06	170.78	172.94	172.98	177.26	41.94	50.78
162.5	163.63	164.67	0.9	172.27	174.21	172.27	176.69	44.09	52.95
165	166.8	167.9	0.71	173.86	175.72	173.86	172.72	42.41	52.2
167.5	169.33	170.24	0.29	174.9	176.92	174.9	176.92	54.49	54.49
170				174.63	178.44	174.63	178.44	52.07	52.07

Table S7: Induction times determined for the isothermal crystallization of the PA12 materials.

T_a	$t_{ind}^{non\text{-}degraded}$	t_{ind}^{mixed}	$t_{ind}^{degraded}$
°C	min	min	min
150	0.21	0.35	0.2
152.5	0.27	0.43	0.28
155	0.24	0.6	0.19
157.5	0.43	1.07	0.49
160	1.04	1.75	0.91
162.5	1.27	4.35	1.27
165	2.62	6.81	3.04
167.5	5.85	7.35	4.8
170	20.83	41.93	18.18

Section S3: Non-isothermal and isothermal crystallization data obtained for the present PA12 materials fit by the MCHL model (points are experimental data; lines correspond to the sc-MKA fits).

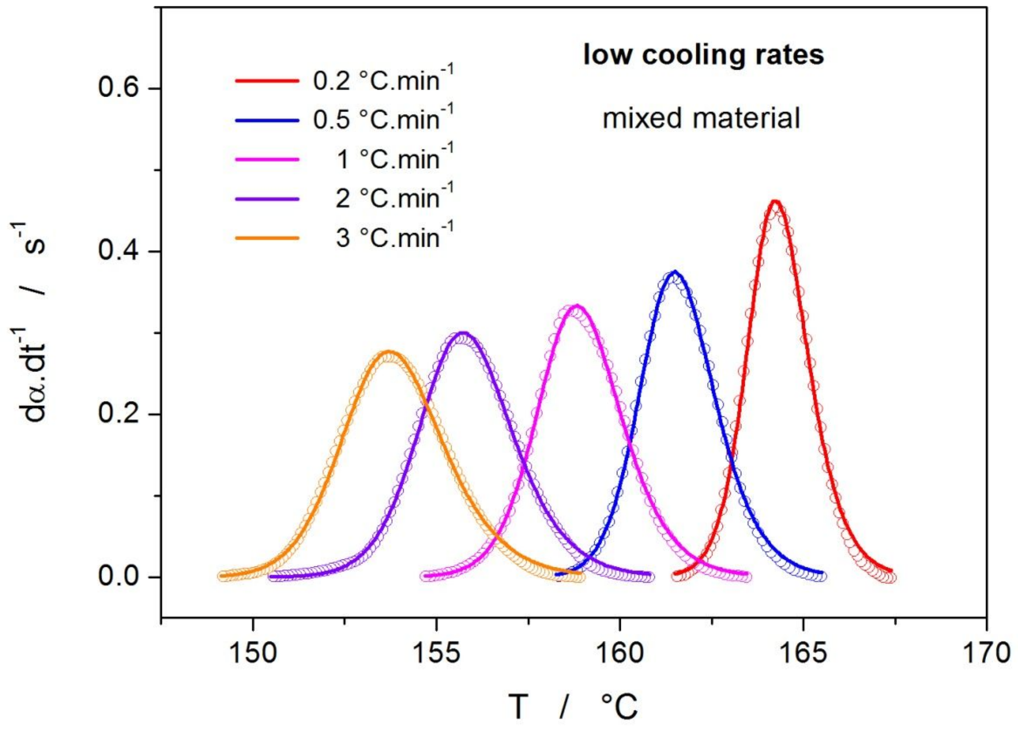

Fig. S6: Data for the mixed material measured at low q⁻.

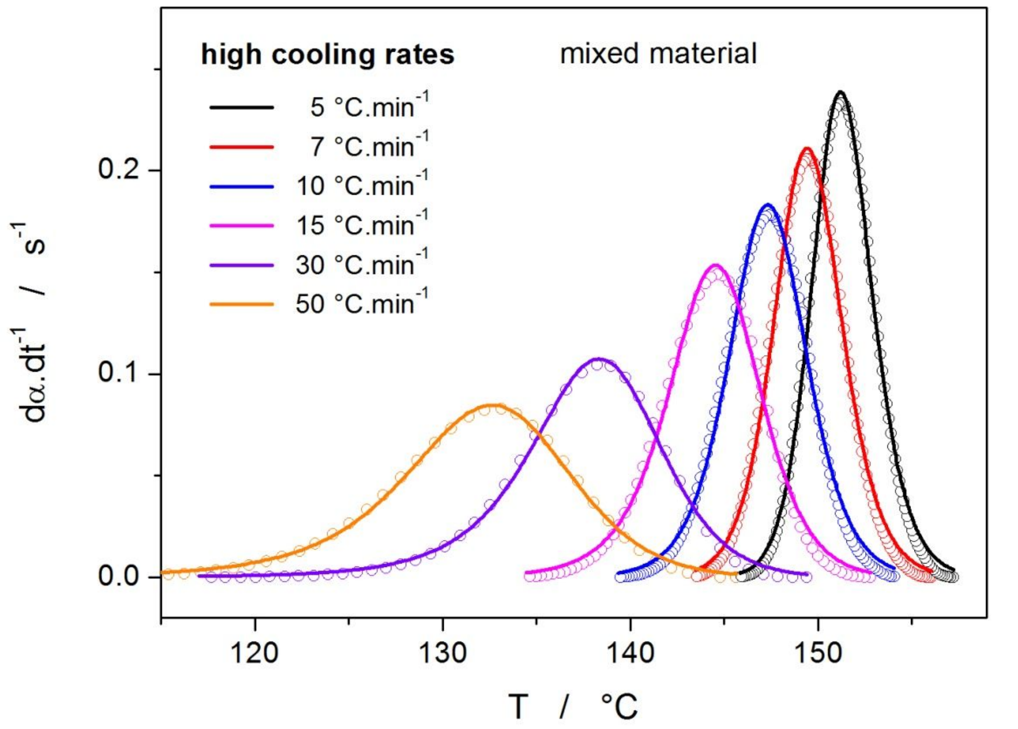

Fig. S7: Data for the mixed material measured at high q⁻.

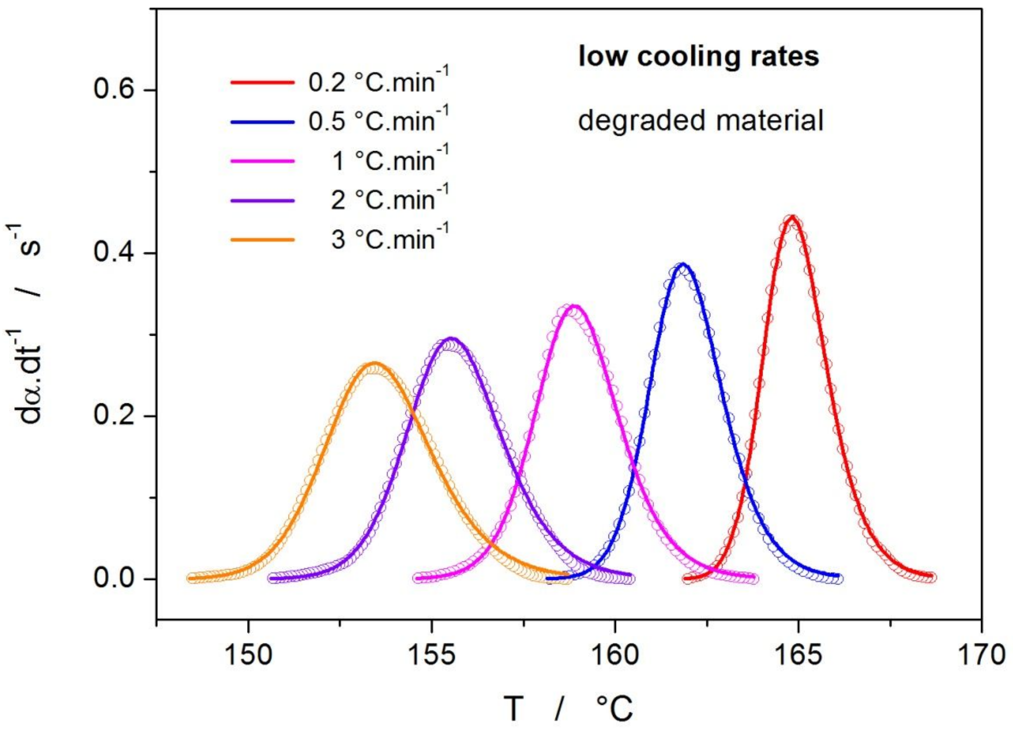

Fig. S8: Data for the degraded material measured at low q^-.

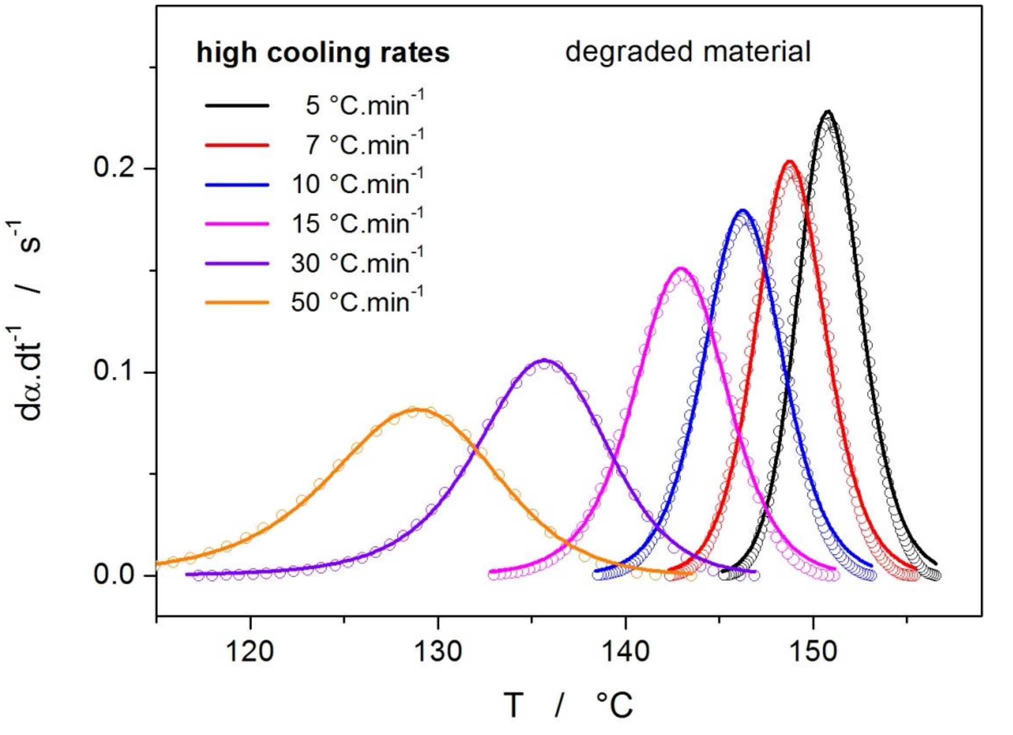

Fig. S9: Data for the degraded material measured at high q^-.

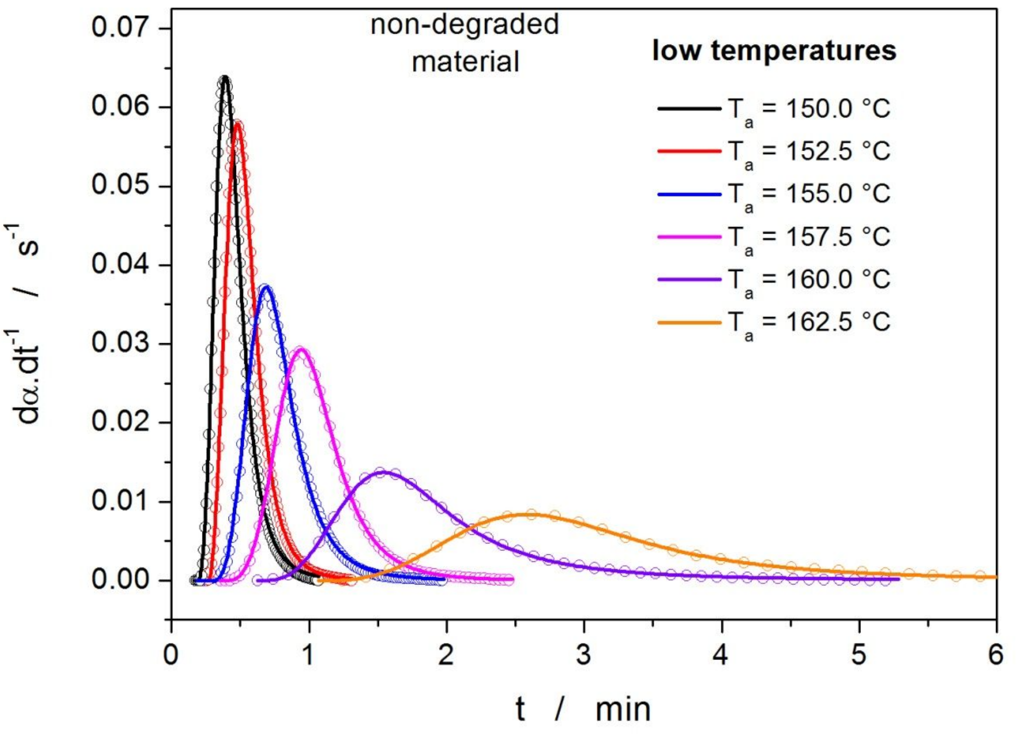

Fig. S10: Data for the non-degraded material measured at low T_a.

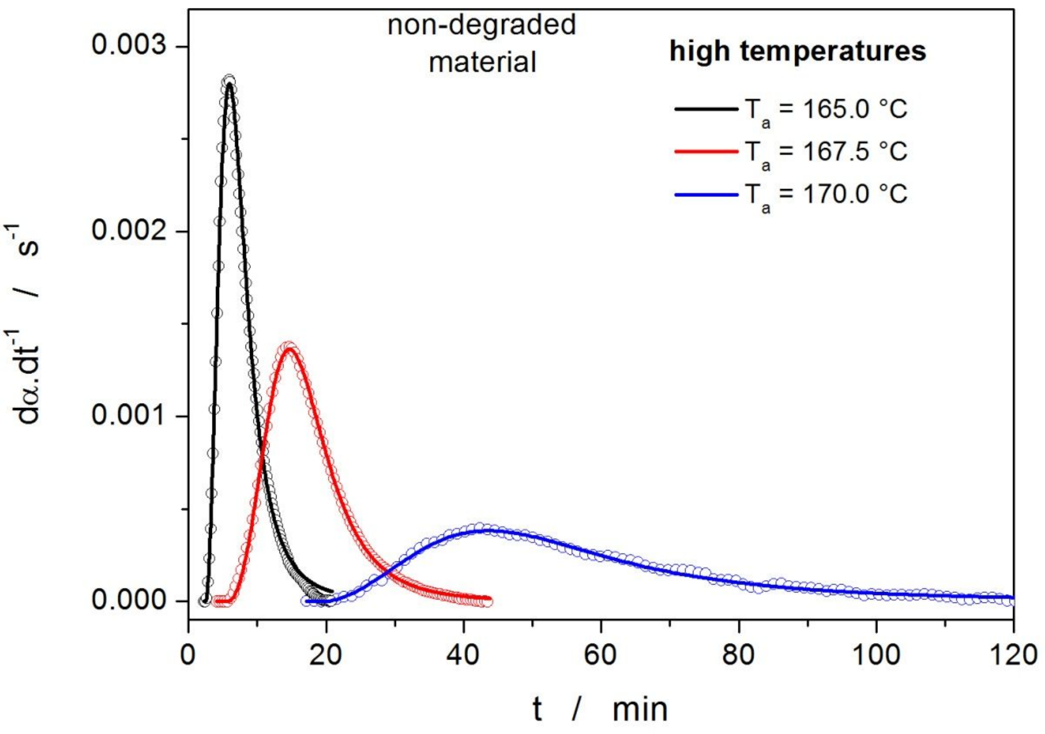

Fig. S11: Data for the non-degraded material measured at high T_a.

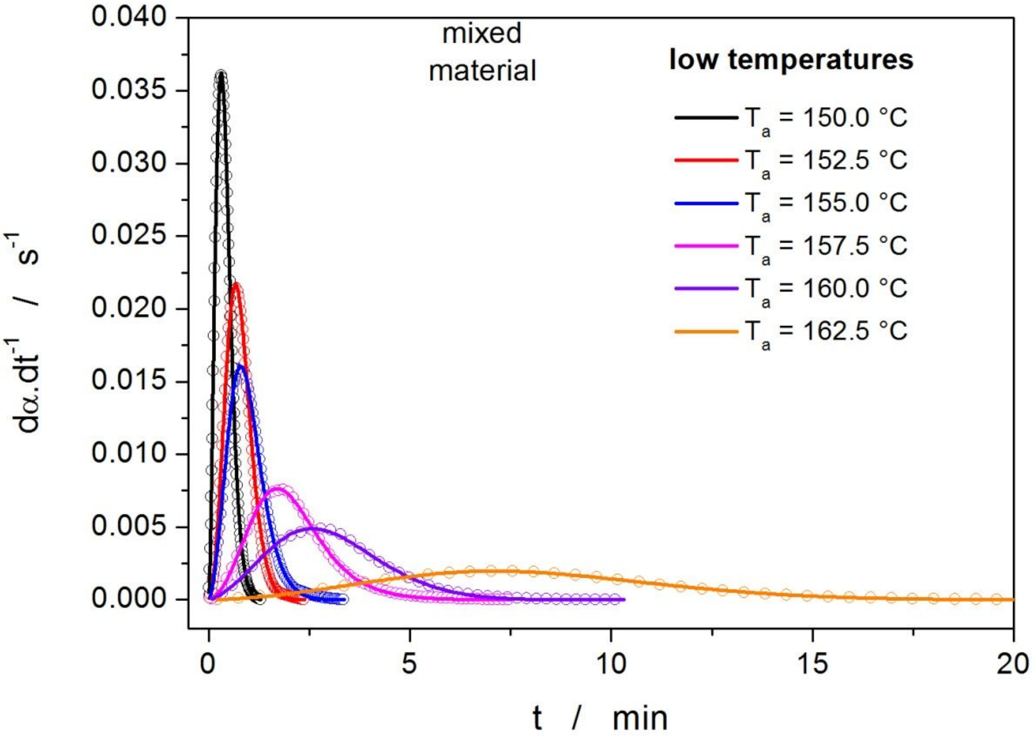

Fig. S12: Data for the mixed material measured at low T_a.

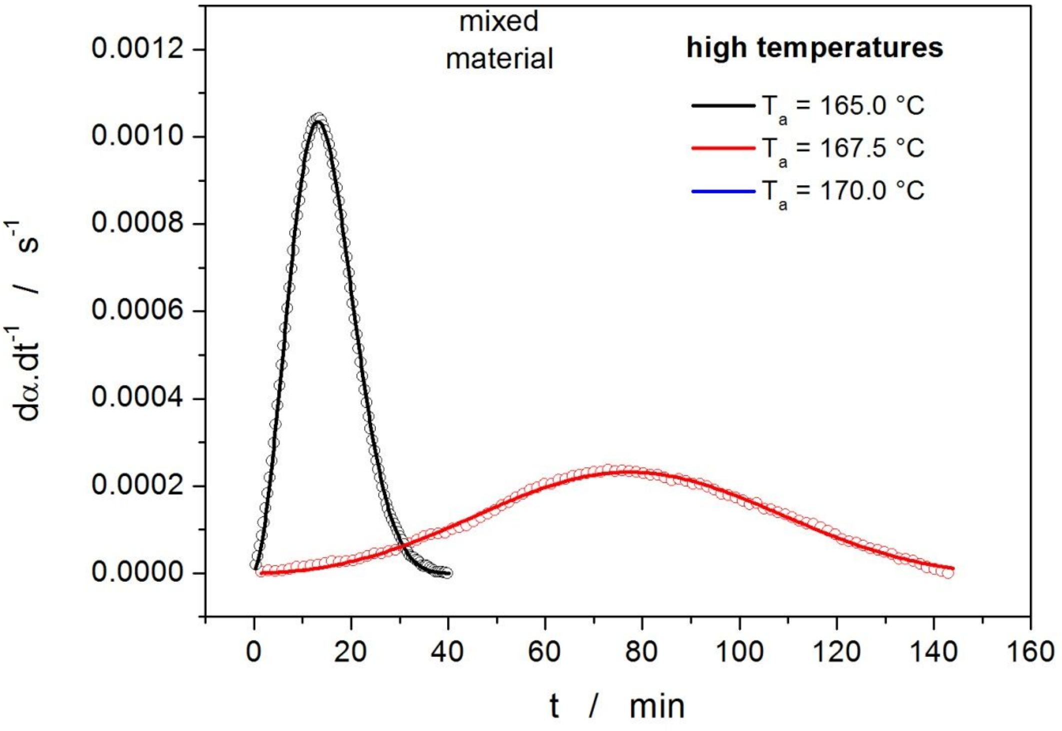

Fig. S13: Data for the mixed material measured at high T_a.

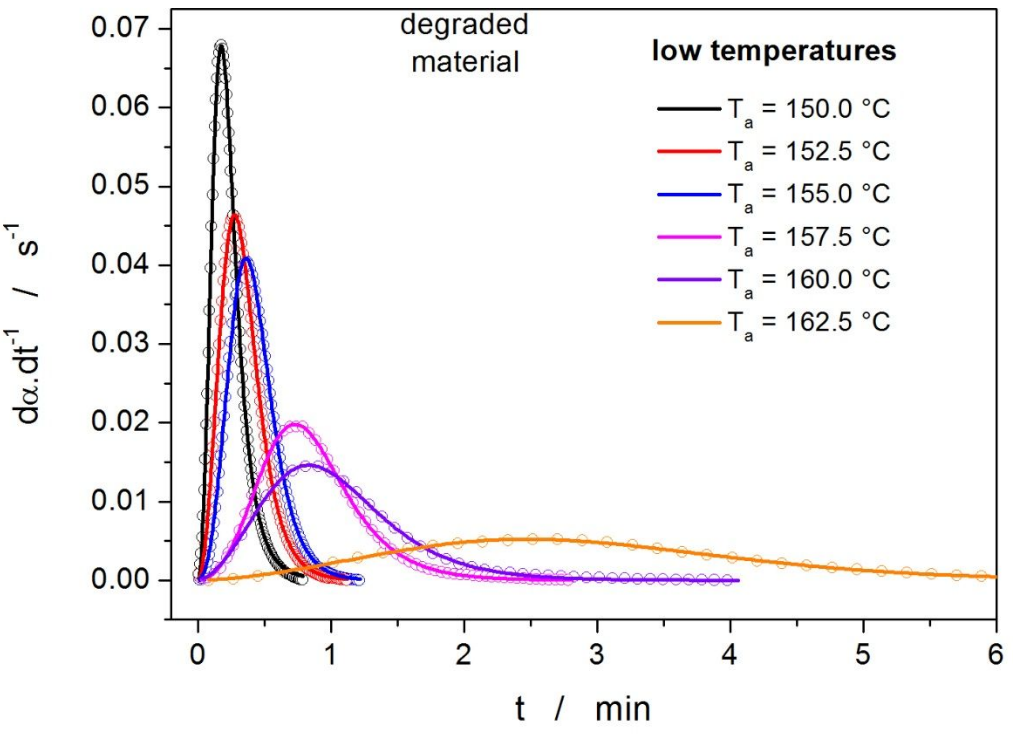

Fig. S14: Data for the degraded material measured at low T_a.

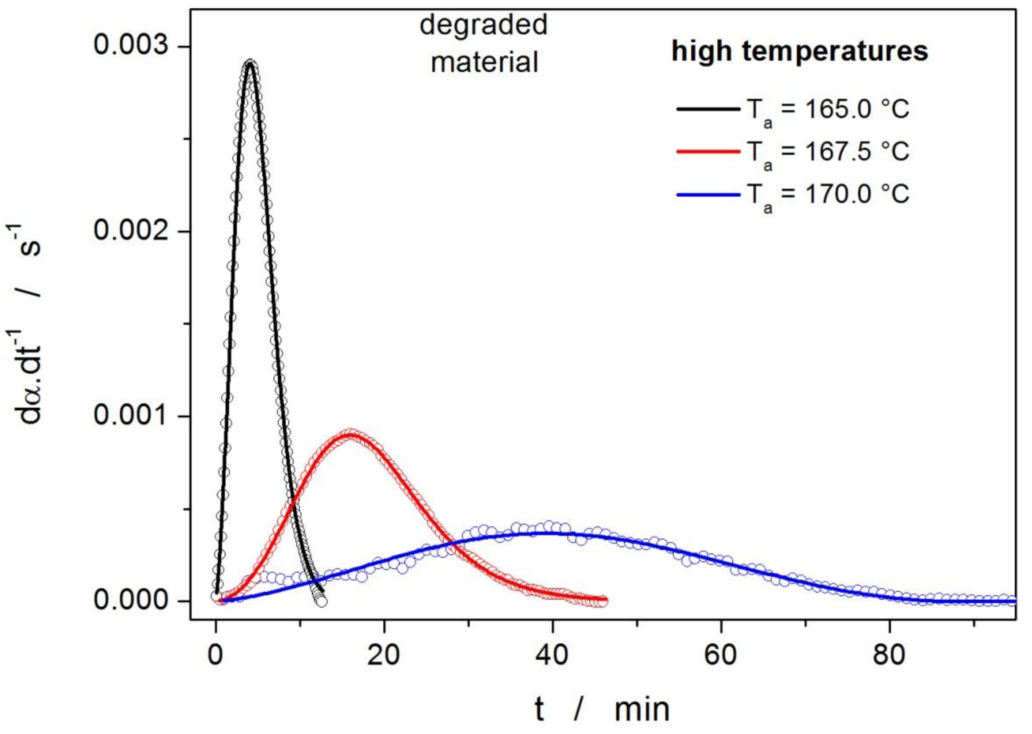

Fig. S15: Data for the degraded material measured at high T_a.
